# A fruit firmness QTL identified on linkage group 4 in sweet cherry (*Prunus avium* L.) is associated with domesticated and bred germplasm

**DOI:** 10.1038/s41598-019-41484-8

**Published:** 2019-03-21

**Authors:** Lichun Cai, José Quero-García, Teresa Barreneche, Elisabeth Dirlewanger, Christopher Saski, Amy Iezzoni

**Affiliations:** 1 Department of Horticulture, Michigan State University, East Lansing, MI 48824 USA; 2 UMR 1332 BFP, INRA, Université de Bordeaux, F-33140 Villenave d’Ornon, France; 3 Department of Plant and Environmental Sciences, Clemson University, Clemson, SC 29634 USA

## Abstract

Fruit firmness is an important market driven trait in sweet cherry (*Prunus avium* L.) where the desirable increase in fruit firmness is associated with landrace and bred cultivars. The aim of this work was to investigate the genetic basis of fruit firmness using plant materials that include wild cherry (syn. mazzard), landrace and bred sweet cherry germplasm. A major QTL for fruit firmness, named *qP-FF4.1*, that had not previously been reported, was identified in three sweet cherry populations. Thirteen haplotypes (alleles) associated with either soft or firm fruit were identified for *qP-FF4.1* in the sweet cherry germplasm, and the “soft” alleles were dominant over the “firm” alleles. The finding that sweet cherry individuals that are homozygous for the “soft” alleles for *qP-FF4*.1 are exclusively mazzards and that the vast majority of the bred cultivars are homozygous for “firm” alleles suggests that this locus is a signature of selection. Candidate genes related to plant cell wall modification and various plant hormone signaling pathways were identified, with an expansin gene being the most promising candidate. These results advance our understanding of the genetic basis of fruit firmness and will help to enable the use of DNA informed breeding for this trait in sweet cherry breeding programs.

## Introduction

Sweet cherry (*Prunus avium* L.) is an important fruit crop in temperate regions and fresh fruit is highly valued. Sweet cherries for the fresh market are hand harvested, often mechanically sorted and frequently in transit for several weeks to distant markets. Because of the harvesting, handling and marketing practices, fresh market sweet cherries need to be firm when harvested. Consumers also prefer fresh market sweet cherries that are firm^1^. Sour cherry (*Prunus cerasus* L.), a tetraploid relative of the diploid sweet cherry, is primarily used for processed products such as jam, juice and pie filling, and therefore neither the supply chain nor the consumer requires the level of firmness necessary for sweet cherry. In addition, the softer texture of sour cherry is a positive attribute for their use in cooked products and beverages. However, very soft sour cherries are rejected by the processors as mechanical pit removal is problematic.

The sweet cherries grown for fruit production were domesticated from wild cherry (syn. mazzard), which is believed to have originated around the Caspian and Black Seas and subsequently spread throughout Europe and south to Iran^2^. Mazzards have extremely small fruit and are grown for their high value lumber. The three mazzards used in this study also have very soft fruit. Increases in fruit size and firmness are the main fruit traits associated with the domestication of sweet cherry from its wild relatives. Sour cherry was formed from the hybridization between sweet cherry and the tetraploid ground cherry (*P. fruticosa* Pall.), a wild bush species native to Eastern Europe^2^. Sour and ground cherry have fruit that is significantly softer than that of the commercial sweet cherry.

The importance of fruit firmness for sweet cherry can be traced back to early records of cherry cultivation. For instance, sweet cherry cultivation in the Jerte Valley, Extremadura, Spain, reported for the first time in 1352, was based on farmer’s selection of local cultivars with improved quality attributes, including firmer fruit^3^. Cherry cultivation in this region grew significantly during the XIX^th^ century, and was mainly based on four very firm cultivars harvested stemless, which were traditionally known as ‘Picotas’ including ‘Ambrunés’, ‘Pico Negro’, ‘Pico Limon Negro’ and ‘Pico Colorado’. At a period when no modern transportation systems existed in Spain, cherries were transported with mules from this valley to the country’s capital, Madrid, a several day journey, and consumer demand suggested that the fruit still kept acceptable quality. Another example of a relatively old firm cultivar is ‘Bing’, which was selected from a seedling of ‘Black Republican’ in 1875 in Oregon, USA^4^. Today, ‘Bing’ still remains the most important cultivar of the Pacific Northwest, USA, and has also been fundamental in the extremely fast development of sweet cherry cultivation in Chile, which is a country that directs most of its production to long-distance export markets. One of the first goals of sweet cherry breeding programs was to select hybrids with large and firm fruits. As an example, the INRA breeding program released during the 1980’s the cultivar ‘Fercer’ (Arcina®), obtained from an open pollination of ‘Stark Hardy Giant’, which was one of the first cultivars producing very large fruit (up to 15 g) with a high level of firmness as well. Subsequently, this cultivar was heavily used as a parent in the INRA breeding program, leading to many new cultivars, such as ‘Folfer’, ‘Ferdouce’, ‘Fertille’ or ‘Ferdiva’^5^.

Little is known about the genetic control of fruit firmness in either sweet or sour cherry. In a study of two F_1_ populations derived from three sweet cherry cultivars (‘Regina’ × ‘Lapins’, ‘Regina’ × ‘Garnet’), the phenotypic data of the progeny fit normal distributions suggesting that the trait was quantitatively inherited^6^. Multiple quantitative trait loci (QTLs) for firmness were identified, the largest one on linkage group (LG) 5, but none of the QTL explained more than 24.1% of the phenotypic variance. A second QTL study was done using the sweet cherry population ‘Ambrunés’ × ‘Sweetheart’ that also exhibited a continuous distribution for firmness^7^. In this case, a previously undetected QTL was identified on LG 1 along with a previously identified QTL on LG 6. No QTL for fruit firmness have yet to be identified in sour cherry.

Genes controlling fruit firmness have been well investigated in many species including tomato, peach and apple. In these species, the physiological modifications of the cell wall organization were considered important components of tissue firmness^8^. Many enzymes, which are capable of altering cell wall texture, have been proposed^9,10^. For example, endopolygalacturonase (endoPG), encoded by a multiple-gene family, is well established as one of the major enzymes involved in pectin disassembly in tomato and kiwifruit^11,12^. In apple, endoPG was also shown to be involved in fruit softening process and its regulation was found to be ethylene dependent^13^. Copy number variation of a gene cluster encoding endoPG was also found to mediate flesh texture in peach^14^. In sweet cherry, genome-wide transcriptional dynamics from developing fruit between flowering and maturity at 14 time points were investigated and the results suggested tight developmental regulation of genes functioning in diverse processes such as sugar transport, lipid metabolism and cell wall rearrangement related to changes in fruit firmness^15^. To date, no genes have been identified that control the variation for fruit firmness in sweet cherry, but candidate genes underlying the firmness QTL identified on LG 5 in sweet cherry have been proposed^6^. In sour cherry, expansin genes were found to be upregulated during ripening (also the period of fruit softening)^16^.

The objectives of this study were to (1) identify and characterize the QTL(s) for fruit firmness segregating in an F_1_ sweet cherry population, (2) explore whether the QTL identified is associated with the firmness that accompanied sweet cherry domestication and breeding, and the presence of softer fruit exhibited by sour cherry, and (3) identify candidate genes for fruit firmness within the QTL region.

## Results

### Phenotypic variation for fruit firmness

**Figure 1:**
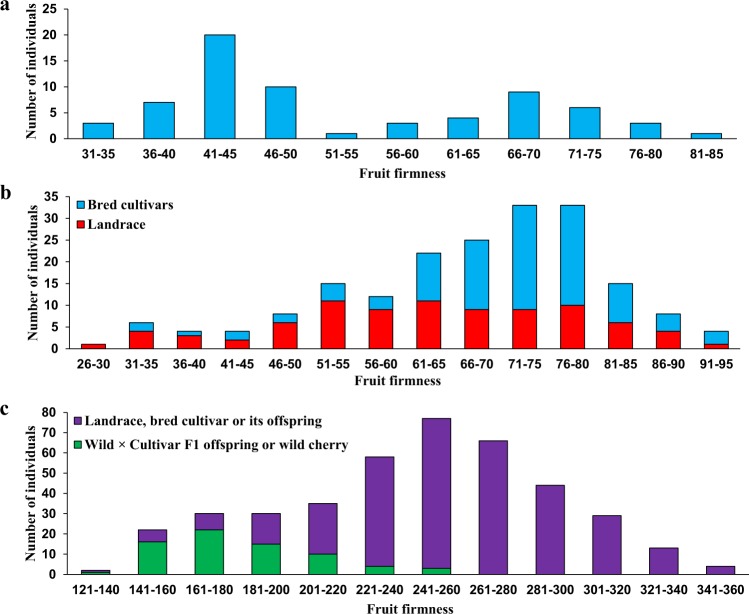
Frequency distributions of fruit firmness for three sweet cherry panels. (**a**) INRA ‘Fercer’ × ‘X’ F_1_ population; (**b**) INRA germplasm collection; (**c**) RosBREED pedigreed germplasm. Data from 2015 was used for (**a**,**b**) since it is the only common year in which firmness data was collected for the two INRA sweet cherry populations. Data summaries and frequency distributions using multi-year data are presented in Table 1 and Fig. S1, respectively.

The progeny in the ‘Fercer’ × ‘X’ population exhibited a wide range of fruit firmness; however, the distribution was bimodal with more individuals exhibiting soft fruit (Table 1, Figs 1a and S1). ‘X’ was assigned as the paternal parent of this population since the recorded paternal parent was found incorrect based on genotype data and the correct parent is unknown. ‘Fercer’ had a multi-year fruit firmness mean of ~67 g/mm^2^ (Min 56, Max 82) which is aligned with the firm-fruited progeny group. A wide range of variation for fruit firmness was also observed for individuals from the INRA sweet cherry germplasm collection and RosBREED germplasm (Fig. 1b,c). In the INRA sweet cherry germplasm collection, the majority of soft-fruited individuals were characterized as landraces as opposed to bred cultivars. In the RosBREED germplasm, the majority of soft-fruited individuals were either mazzards or hybrids with mazzards. When the two INRA populations were compared for firmness for the one year that they were both phenotyped, the sweet cherry germplasm collection exhibited a wider phenotypic distribution compared to the F_1_ population (Fig. 1). Within the three populations, the ANOVA analysis revealed highly significant effects for the different genotypes (Table 1). In all three populations, the broad-sense heritabilities were high (0.73–0.97), indicating that much of the phenotypic variation in these populations is genetically controlled (Table 1). The highest heritability for fruit firmness (0.97) was obtained from the multi-year data for the ‘Fercer’ × ‘X’ population, likely because the environmental variation was low among years compared to the genetic variation as suggested by the bimodal phenotypic distribution.

**Table 1:** Summary of fruit firmness data for three sweet cherry populations.

Population^a^	Size	Mean ± SD	Range	σ^2^_g_	σ^2^_e_	Heritability
INRA ‘Fercer’ × ‘X’ F_1_ population^b^	67	55 ± 12	32–80	139	30	0.97
INRA germplasm collection^c^	193	65 ± 11	35–86	95	72	0.73
RosBREED pedigreed population^d^	528	243 ± 47	137–351	2535	244	0.91

Note: ^a^Different methods for phenotyping were used for the INRA and RosBREED populations. ^b^Phenotypic data is from seven years (2009–2013, 2015–2016). ^c^Phenotypic data is from two years (2014–2015). ^d^Phenotypic data is from two years (2011–2012).

The firmness of the RosBREED sweet cherry materials and sour cherry materials could be directly compared as they were phenotyped using the same instrumentation. Fruit from the sour cherry individuals exhibited a smaller range of firmness (98–197) compared to sweet cherry (137–397) (Figs 1 and S2). The fruit firmness of 95% of the sour cherry individuals was less than 170, indicating that almost all the sour cherries were softer than the sweet cherry landraces, bred cultivars, and their offspring.

### QTL analysis

**Figure 2:**
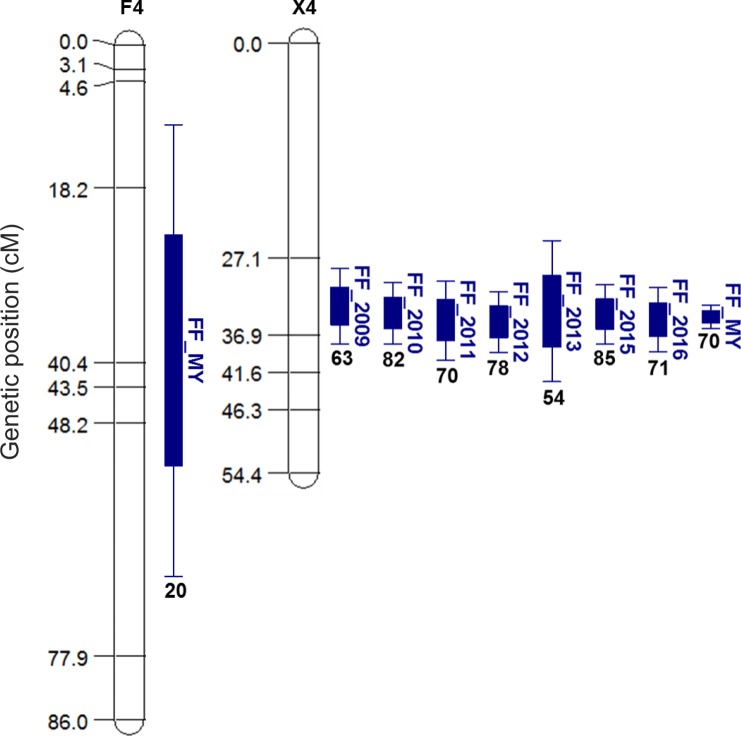
Summary of QTLs for fruit firmness (FF) identified in the INRA ‘Fercer’ × ‘X’ F_1_ population. QTLs are shown for each year when detection was significant as well as by combining multiple years of data (MY) using the ‘multi-environment’ option of MultiQTL software. Explained percentage of variance is given for each QTL.

*‘Fercer’* × *‘X’*: The two parental maps constructed consisted of 110 SNPs for ‘Fercer’ and 87 SNPs for ‘X’ with an average coverage of one marker every 6.7 and 7.5 cM, respectively. QTL analysis from the ‘Fercer’ × ‘X’ population identified four QTL in the multi-year analysis (LGs 4, 5, 6 and 8); however, the major stable QTL was located on LG 4 (Table 2, Fig. 2 and Table S1). This QTL segregating from the ‘X’ parent was significant in all seven years evaluated and across years, and the percentage of variation explained by the QTL ranged from 54.0% to 84.6%. The QTL confidence interval based on multiple years’ analysis was small (33.1–36.0 cM) and estimated to only cover about one Mbp (10,335,393–11,216,807 bp) based on the peach physical map v.2. The peak position of this QTL was located at 34.5 cM, which was estimated to be approximately 10.76 Mbp on the peach physical map v.2 (Table 2). One QTL was identified segregating from ‘Fercer’, but this QTL was only significant over multiple years, explaining 20.1% of the phenotypic variance. As the QTL confidence intervals overlapped, the QTLs derived from ‘X’ and ‘Fercer’ were considered to be the same and this QTL was named *qP-FF4.1*.

**Table 2:** Summary of QTLs for fruit firmness identified on linkage group (LG) 4 in the ‘Fercer’ × ‘X’ F_1_ population.

Year	LG^a^	LOD^a^	CI (cM)^a^	Peak (cM)	D^a^	PVE (%)^a^
2009	X^a^4	10.7	28.4–38.0	33.2	−17.6	62.8
2010	X4	23.3	30.2–38.0	34.1	−26.3	82.4
2011	X4	18.1	30.0–40.1	35.0	−22.5	70.2
2012	X4	22.4	31.4–39.1	35.2	−22.9	78.3
2013	X4	11.3	24.9–42.8	33.9	−18.7	54.0
2015	X4	25.9	30.5–38.0	34.2	−25.7	84.6
2016	X4	17.6	30.8–39.0	34.9	−21.7	70.8
MY^b^	F^a^4	20.6	10.3–67.7	39.0	−12.1	20.1
MY^b^	X4	125.3	33.1–36.0	34.5	−21.8	70.2

Note: ^a^Linkage group (LG), LG of ‘X’ (X), LG of ‘Fercer’ (F); Logarithm of odds ratio (LOD); Confidence interval (CI); Difference between the two homozygotes at the marker loci (d); Percentage of variation explained by the QTL (PVE). ^b^Significant QTLs are presented for each year as well as significant QTL identified when data was combined over multiple years (MY).

**Figure 3:**
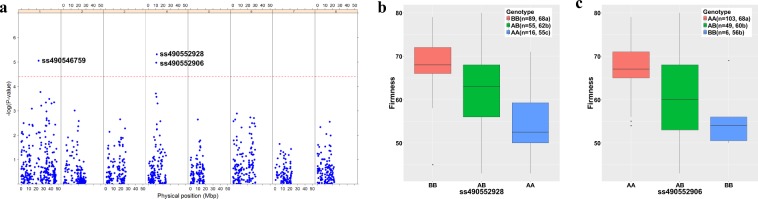
Association of genome-wide SNP markers with fruit firmness in the INRA sweet cherry germplasm collection. (**a**) P values for all the SNP markers across the sweet cherry genome. The red dash line indicates the threshold value for significant SNPs after Bonferroni correction (0.05/1215). (**b**) Phenotypic means based on the genotype of the most significant SNP marker ss4905522928, and (**c**) the second most significant SNP marker ss490552906. Phenotypic means that were significantly different (P < 0.05) are identified by different letters.

*INRA Sweet Cherry Germplasm Collection:* In the INRA sweet cherry germplasm collection, variation in fruit firmness significantly associated with SNPs located on chromosome four (Fig. 3a). The SNP most significantly associated with fruit firmness, ss490552928, has a peach physical map position (v.2) of 11,472,398 bp. Fruit firmness was significantly different among the three SNP genotypes, as illustrated for ss490552928 and the second most significant SNP on chromosome 4, ss490552906, located at 10,880,163 bp (Fig. 3b,c). For ss490552928, mean fruit firmness for the SNP genotype AB, was intermediate to that of BB and AA, with increased firmness associated with BB (Fig. 3b). For ss490552906, mean fruit firmness for the SNP genotype AB was significantly less than that of the most firm genotypic class (AA) and not significantly different from the softest class (BB) (Fig. 3c). In addition to the SNPs on chromosome 4, an additional SNP on chromosome 1 (ss490546759, 23,455,434 bp) was significantly associated with fruit firmness (Fig. 3a). This SNP is within a region where a fruit firmness QTL was previously identified in four of six years in a ‘Regina’ × ‘Lapins’ population^6^.

*RosBREED Sour cherry germplasm*: The sour cherry germplasm was also segregating for ss490552928; however, no sour cherry individuals had the BBBB genotype (Fig. S3a). The majority of the individuals were AAAB, followed by AAAA, AABB and then ABBB. The association of SNP genotype and fruit firmness was investigated within two segregating sour cherry populations and found to be not significant (Fig. S3b,c). This is in contrast to the INRA sweet cherry germplasm collection where BB was the most prevalent genotype for this SNP and BB individuals had significantly firmer fruit than AB or AA individuals (Fig. 3b).

**Figure 4:**
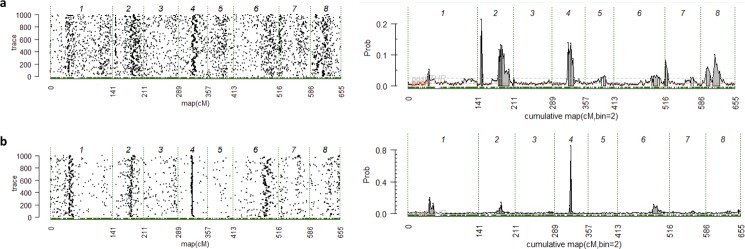
QTL mapping result for RosBREED sweet cherry population illustrated by trace plots and posterior probability of QTL positions along the genome exported from software FlexQTL. The beginning and the end of the linkage groups are represented by vertical dashed lines. The solid gray areas on the right side correspond to regions with positive evidence for the presence of QTLs. (**a**) Fruit firmness in 2011; (**b**) Fruit firmness in 2012.

*RosBREED sweet cherry germplasm*: Two fruit firmness QTLs were identified in both years for the RosBREED pedigree germplasm, one small effect QTL on LG 2 and one large effect QTL on LG 4 (Table 3, Fig. 4 and Table S1). The QTL on LG 4 explained 16.4% and 83.5% of the variation for fruit firmness in 2011 and 2012, respectively. This difference is probably due to the high number of missing values in 2011 compared to 2012 (219 vs. 126, respectively). The peak genetic map position of this QTL was 33 cM and the peak physical map position was estimated to be ~10.8 Mbp. As this is similar to the peak position of the QTL identified in the ‘Fercer’ × ‘X’ population, this QTL was considered to be *qP-FF4.1*. Predictions for the genotypes of *qP-FF4.1* for the RosBREED germplasm were calculated by FlexQTL, as *QQ*, *Qq* and *qq*, where *Q* and *q* represent the “firm” and “soft” alleles, respectively. FlexQTL used a bi-allelic model denoted by *Q* and *q* to estimate the QTL genotypes. The only individuals in this germplasm set predicted to be *qq* (and therefore soft) were three mazzard accessions, MIM 17, MIM 23, and NY 54 (Table S2). Likewise the only individuals in this germplasm set predicted to be *Qq* were offspring from these three mazzard accessions plus ‘Moreau’ and ‘Cristobalina’, old landrace cultivars, and their offspring. All other individuals were predicted to be *QQ*. This suggests that homozygosity for the firm *Q* allele at *qP-FF4.1* is a signature of selection exhibited by domesticated and bred sweet cherries.

**Table 3:** Summary of the fruit firmness QTL identified on linkage group (LG) 4 in the RosBREED germplasm.

Year	Interval (cM)	Peak (cM)	BF^a^	Peak (cM)	Effect^a^	PVE (%)^a^
2011	31–47	33	4.8	33	48	16.4
2012	31–37	33	32.1	33	83	83.5

Note: ^a^Significance was presented by Bayes Factor (BF); Additive effect (Effect); Percentage of variation explained by the QTL (PVE).

### Haplotype analysis

**Figure 5:**
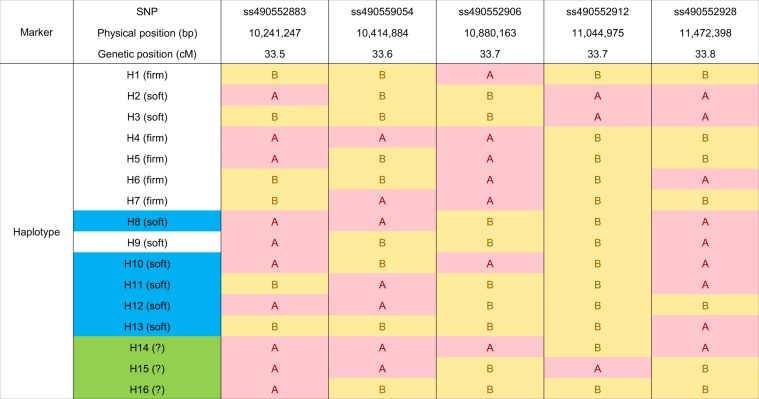
Haplotypes for the fruit firmness QTL, *qP-FF4.1*, and their physical positions on the peach Genome v2.0^39^. In sweet cherry, haplotypes only identified in mazzard are marked as blue. Haplotypes only identified in sour cherry are marked as green. The full list of plant materials exhibiting the 16 haplotypes are listed in Table S3. The haplotypes for eight sour cherry parents are presented in Table S4. The haplotypes were deduced to be associated with soft or firm fruit based on diplotype analysis in sweet cherry (Fig. 6).

To further trace and evaluate the allele effects of *qP-FF4.1*, five SNPs that span the peak physical map QTL location were chosen for haplotype (allele) construction (Fig. 5). These five SNPs spanned a ~1.23 Mbp and ~0.3 cM region of LG 4. Using these five SNPs, 13 haplotypes (H1 to H13) were identified in the RosBREED sweet cherry germplasm and an additional three haplotypes were identified in sour cherry (H14 to H16) (Tables S3 and S4). As the QTL haplotypes were based on SNP marker composition spanning the QTL region and not variation in underlying genes, it is possible that the haplotypes identified over-represent the number of functional alleles. Of the thirteen haplotypes exhibited by sweet cherry, four were only identified in the mazzards (H8, H11, H12 and H13) (Tables S2, S3). The haplotypes most frequent in the RosBREED sweet cherry germplasm and also not present in any mazzards were H4 (49.1%) and H1 (28.2%), suggesting that these haplotypes are associated with firm fruit possibly due to the influence of human selection and breeding. However, as only three mazzards were used in this study, it is possible that other mazzards might also possess a “firm” allele. Two of the commercially dominant cultivars notable for their fruit firmness, ‘Bing’ and ‘Ambrunés’, are H1H1 and H4H4, respectively. The haplotypes most frequent in the sour cherry selections were H3 (46.9%) followed by H11 (18.8%) and H10 (12.8%).

**Figure 6:**
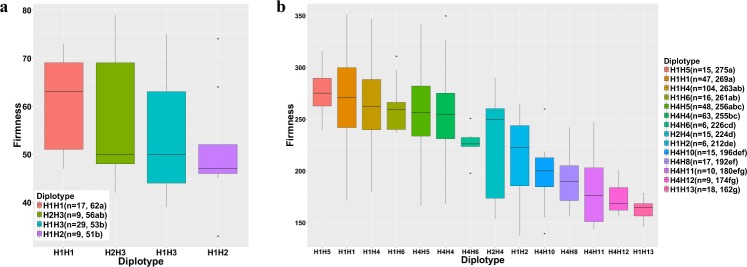
Comparison of fruit firmness (g/mm^2^) for progeny based on their diplotypes for *qP-FF4.1* from (**a**) the ‘Fercer’ × ‘X’ F_1_ population; and (**b**) the RosBREED pedigreed population. Only diplotypes represented by six or more individuals were included. Phenotypic means that were significantly different (P < 0.05) are identified by different letters.

The *qP-FF4.1* genotypes (diplotypes) for ‘Fercer’ and ‘X’ were H1H2 and H1H3, respectively (Fig. 6a). As ‘Early Burlat’ is the only sweet cherry founder known to have H3, it is likely that ‘Early Burlat’ is an ancestor of ‘X’. When the fruit firmness of the ‘Fercer’ × ‘X’ progeny were compared based on their *qP-FF4.1* diplotypes, those progeny that were H1H1, had significantly firmer fruit than progeny that were H1H3 or H1H2 (Fig. 6a). This is consistent with the high relative frequency of H1 in bred germplasm. Furthermore, it suggests that H1 is recessive to H3 and H2. In other words, for this QTL, firm fruit appears to be recessive to soft fruit. H1 and H2 were deduced to be “firm” and “soft” alleles, respectively, as H1H2 individuals had significantly softer fruit than H1H1 individuals. The inheritance of H3, uniquely present in ‘Early Burlat’ and not present in any other RosBREED germplasm, was further followed through breeding using this germplasm. Five U.S. cultivars have ‘Early Burlat’ in their ancestry and all five inherited the ‘Early Burlat’ H6, and not H3 (Fig. S4).

The effects on fruit firmness associated with 14 *qP-FF4.1* diplotypes were compared for the RosBREED germplasm. These 14 diplotypes, representing 10 haplotypes (H1, H2, H4 to H6, H8, H10 to H13), each consisted of firmness data from six to 104 individuals (Fig. 6b). Fruit firmness ranged from a mean of 275 down to 162 for the softest fruit. The four diplotypes that had the firmest fruit all had one or two copies of H1, paired either with itself or with H4, H5 or H6. This suggests that in addition to H1 and H4, H5 and H6 can also be considered “firm” alleles. However, when H1 or H4 were paired with H2, the mean fruit firmness was reduced significantly. This is consistent with the dominant ‘soft’ effect of H2 observed in the ‘Fercer’ × ‘X’ progeny where H1H2 progeny had significantly softer fruit than H1H1 progeny. Progeny with H8 and H10-13, only present in the mazzards, had significantly softer mean fruit firmness than the majority of progeny homozygous for the firm diplotypes. These effects were based on pairings with the “firm” haplotypes H1 or H4, indicating that these “soft” haplotypes present in wild cherry are dominant to the “firm” haplotypes that are found in bred cultivars. It was not possible to determine if the three haplotypes identified in sour cherry (H14-16) were associated with firm or soft fruit due to the dominance of soft compared to firm fruit (Table S4).

### *In silico* candidate genes

**Figure 7:**
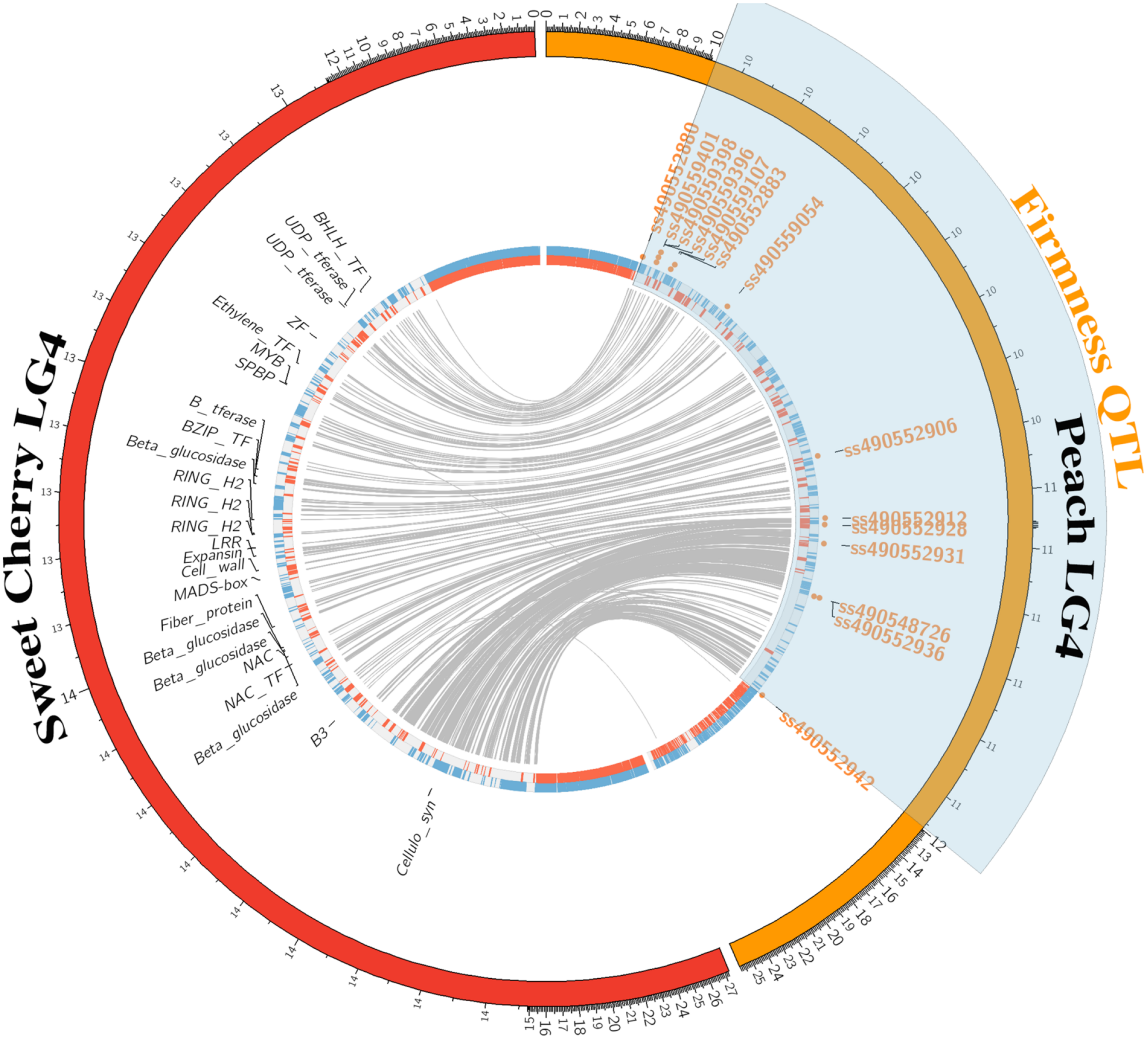
A circos plot depicting linkage group (LG) 4 in the ‘Lovell’ peach reference genome v.2.0 (orange) and the corresponding LG 4 in the sweet cherry genome (red). The firmness QTL region is highlighted in translucent blue and mapped SNP markers as orange circular glyphs. The detailed information for SNP markers in the QTL region is presented in Table S6. The blue and red histograms are annotated genes, with direction of transcription as blue (forward) or red (reverse). Candidate genes are labeled in the sweet cherry region.

The *qP-FF4.1* interval identified from both the INRA F_1_ and RosBREED pedigreed populations was used for candidate gene identification. This ~ 1.8 Mbp interval was between SNPs located at 10,156,468 and 11,956,655 bp on chromosome 4 of the peach genome v2.0 and the same SNPs located between 12,928,603 and 14,860,789 bp on the sweet cherry genome (Fig. 7). In this region, 241 genes were predicted in the sweet cherry genome (Table S5). From these genes, 25 were selected as candidate genes based on their potential to be involved in the control of fruit firmness (Table 4, Fig. 7). The most promising candidate gene identified was Pav_sc0002828.1_g410.1.mk which encodes an expansin protein related to plant cell wall metabolism. This gene is very close to the QTL peak and an expansin gene with homology to Pav_sc0002828.1_g410.1.mk was found to be expressed in sour cherry fruit and associated with tissue softening^16^. Of the three expansin genes identified that were upregulated during softening in sour cherry fruit, the expansin gene *PcEXP4* had the highest similarity to the expansin gene in the sweet cherry genome as evidenced by their placement on a distal lineage, relative to the other sour cherry expansins (Fig. S5a,b). The candidate expansin gene contains two functional domains (Expansin EG45 and Expansin CBD) and three encoded signal peptide regions (H, N, and C) located on the N-terminus region (Fig. S5c). Nine other candidate genes were predicted to encode plant cell wall modifying enzymes which have been found to be potentially involved in regulating fruit firmness in peach and apple^17,18^. Fourteen candidate genes were included as they are potentially involved in various plant hormone signaling pathways well known to be involved in fruit maturation and ripening in non-climacteric and climacteric fruits and in sweet cherry firmness^19,20^. Among these candidate genes, two are predicted to be NAC (NAM/ATAF1, 2/CUC2) transcription factors involved in the ethylene signaling pathway. Of these two genes, Pav_sc0000029.1_g070.1.mk, is a homolog of the peach NAC gene ppa008301m that has been predicted to control maturity date^21,22^. The final candidate gene, Pav_sc0000975.1_g210.1.mk, was predicted to be a Squamosa promoter-Binding Protein which has been found to be associated with fruit ripening in tomato^23^.

**Table 4:** Summary of 25 candidate genes for fruit firmness located in the *qP-FF4.1* interval on chromosome 4 in sweet cherry.

Cherry candidate genes	Transcript start and stop (bp)		Description^c^	Biological process
	Cherry genome^a^	Peach genome^a^		
Pav_sc0003492.1_g360.1.br	13090321~13090626	NA^b^	hypothetical protein PRUPE_7G080000*/BHLH transcription factor*^d^	Ethylene signaling pathway
Pav_sc0003492.1_g260.1.mk	13134328~13147715	10270962~10272561	UDP-glycosyltransferase 79B6-like	Plant cell wall metabolism
Pav_sc0003492.1_g250.1.mk	13147752~13148159	10276520~10277987	UDP-glycosyltransferase 79B6-like	Plant cell wall metabolism
Pav_sc0003492.1_g040.1.br	13251924~13253405	NA	Zinc finger MYM-type protein 1-like	Brassinosteroid signaling pathway
Pav_sc0000975.1_g110.1.mk	13329847~13330536	10435004~10436194	Ethylene-responsive transcription factor 4	Ethylene signaling pathway
Pav_sc0000975.1_g200.1.mk	13386372~13389094	10505281~10508243	Probable protein phosphatase 2C29*/MYB transcription factor*	Auxin signaling pathway
Pav_sc0000975.1_g210.1.mk	13389781~13391094	10508967~10510314	Squamosal promoter-Binding Protein 1-like	Fruit ripening regulator
Pav_sc0002828.1_g670.1.mk	13612734~13616541	10718283~10722430	Beta-glucuronosyltransferase GlcAT14B	Plant cell wall metabolism
Pav_sc0002828.1_g650.1.mk	13627727~13637959	10738335~10747338	Probable serine/threonine-protein kinase At1g54610*/BZIP family transcription factor*	Gibberellin signaling pathway
Pav_sc0002828.1_g640.1.mk	13648670~13654523	10755244~10761213	Inner membrane protein oxaA/*Beta-glucosidase*	Plant cell wall metabolism
Pav_sc0002828.1_g510.1.mk	13741324~13741986	10815979~10816956	Putative RING-H2 finger protein ATL19	Brassinosteroid signaling pathway
Pav_sc0002828.1_g470.1.mk	13776148~13776732	10818810~10819608	Putative RING-H2 finger protein ATL19	Brassinosteroid signaling pathway
Pav_sc0002828.1_g460.1.mk	13778460~13779378	10821182~10821971	Putative RING-H2 finger protein ATL19	Brassinosteroid signaling pathway
Pav_sc0002828.1_g410.1.mk	13809261~13815575	10861181~10863415	Expansin-A12	Plant cell wall metabolism
Pav_sc0002828.1_g380.1.mk	13831978~13833770	10882666~10885574	F-box/FBD/LRR-repeat protein At4g26340-like	Gibberellin signaling pathway
Pav_sc0002828.1_g310.1.mk	13868641~13869483	10929344~10930120	Glycine-rich cell wall structural protein 2	Plant cell wall metabolism
Pav_sc0002828.1_g280.1.mk	13892641~13893365	NA	Agamous-like MADS-box protein AGL29	Ethylene signaling pathway
Pav_sc0002828.1_g060.1.mk	14014881~14016967	11036801~11039019	Fiber protein Fb34/*Zinc binding dehydrogenase*	ABA signaling pathway
Pav_sc0000029.1_g020.1.mk	14069597~14074899	11087505~11091386	Beta-glucosidase 18-like isoform X1	Plant cell wall metabolism
Pav_sc0000029.1_g040.1.br	14079090~14079410	11092451~11093649	Beta-glucosidase 18-like isoform X1	Plant cell wall metabolism
Pav_sc0000029.1_g050.1.mk	14080593~14083893	11093783~11095088	Beta-glucosidase 18-like isoform X1	Plant cell wall metabolism
Pav_sc0000029.1_g070.1.mk	14097151~14098925	11116814~11118655	NAC domain-containing protein 72	Ethylene signaling pathway
Pav_sc0000029.1_g090.1.mk	14118138~14120442	11138518~11140641	NAC transcription factor 56	Ethylene signaling pathway
Pav_sc0002607.1_g160.1.mk	14294246~14301322	11300896~11310515	B3 domain-containing protein Os07g0679700-like isoform X1	ABA signaling pathway
Pav_sc0000124.1_g110.1.mk	14575143~14616823	11627434~11632547	Cellulose synthase-like protein G2 isoform X2	Plant cell wall metabolism

Note: ^a^The cherry and peach genome sequences used were from Shirasawa *et al*.^42^ and Verde *et al*.^39^, respectively. ^b^Not available (NA) as no sequence match was found in the peach genome sequence. ^c^The description is the Blast2GO ontology term. ^d^The tomato annotation was included in italic when it differed from the cherry and/or peach annotation(s) and suggested a potential involvement with fruit firmness.

## Discussion

### Genetic determinism and signature of selection for fruit firmness in sweet cherry

The bimodal segregation for fruit firmness in the ‘Fercer’ × ‘X’ population provided the opportunity to identify a major QTL for fruit firmness in sweet cherry that was also identified in a wide range of genetic backgrounds represented by the INRA sweet cherry germplasm collection and the RosBREED pedigreed population. This is the first report of a major QTL for fruit firmness identified on LG 4 in sweet cherry. However, due to the small size of the ‘Fercer’ × ‘X’ population, the QTL interval would be affected by potential errors in phenotyping and genotyping as well as the environmental conditions. Despite this population size limitation, the QTL region estimated for all seven years was stable and consistent, possibly due to the large effect of this QTL in this population as suggested by the bimodal phenotypic distribution. However, future fine mapping is needed to more precisely define the QTL interval. In a prior study of two sweet cherry populations between bred cultivars, ‘Regina’ × ‘Lapins’ and ‘Regina’ × ‘Garnet’, QTLs for fruit firmness detected in at least three of the six years of study, were identified on LG 1, 2 and 5^6^. On LG 4, a QTL was detected in only two of the six years analyzed in one of the two populations (‘Regina’ × ‘Lapins’), and this QTL was located on the upper region of chromosome 4 in a region that does not overlap with that for *qP-FF4.1*. It is possible that the LG 4 QTL, *qP-FF4.1*, was not identified in these two populations, because all three parents only had “firm” alleles for this locus. Indeed, all three haplotypes present in ‘Regina’ (H4H5) and ‘Lapins’ (H1H4) were identified as “firm” alleles in this study. In contrast, the plant materials used in this study resulted in the identification of *qP-FF4.1* because of the presence of “soft” alleles in the plant materials.

The finding that sweet cherry individuals that are homozygous for the “soft” alleles for *qP-FF4*.1 are exclusively mazzards and that the vast majority of bred cultivars are homozygous for “firm” alleles suggests that this locus was a signature of selection during domestication and modern breeding. In addition, three of the old cultivars included in this study, ‘Moreau’, ‘Cristobalina’ and ‘Early Burlat’, have relatively soft fruit and their *qP-FF4.1* genotypes include one “soft” and one “firm” allele. In sour cherry, all of the germplasm are soft, as firmness comparable to sweet cherry has not been a critical trait. This QTL region is the second region in sweet cherry that has been shown to have been under selection, the first being a QTL region on LG 2 that contains a major QTL for fruit size^24^.

The results from the ‘Fercer’ × ‘X’ population and the RosBREED germplasm further indicate that the “soft” alleles present in the mazzard accessions are dominant, or at least partially dominant over the “firm” alleles present in bred cultivars. This is consistent with the findings in sour cherry where no individuals exhibited the firmness of bred sweet cherry cultivars. No sour cherry individual was homozygous for the “firm” allele at *qP-FF4.1*, and therefore all the sour cherry individuals had at least one “soft” allele for *qP-FF4.1*. These results are also consistent with the results from progeny from a cross between a sweet cherry cultivar ‘Emperor Francis’ and the mazzard accession NY 54. A major QTL for fruit size associated with domestication was identified in this F_1_ population^25^; however, QTL for fruit firmness could not be identified from this population because all the progeny had soft fruit. Given that the *qP-FF4.1* diplotypes for ‘Emperor Francis’ and NY 54 are H1H1 and H13H13, respectively, and the conclusion that soft fruit is dominant over firm fruit, this result would be expected.

In peach, a major QTL for fruit firmness was also identified on LG 4, first reported by Dettori *et al*.^26^. This locus, termed *F-M*, controls both peach fruit firmness and flesh adhesion to the endocarp, with soft (melting) fruit dominant to firm (non-melting) fruit. Two genes encoding endopolygalacturonase (endoPG) are considered to be the causal genes at this locus. The peach physical map (v.2.0) positions of these two genes are as follows: ppa006839m 19046344-19049605 bp, and ppa006857m 19081325-19083984 bp. Using the peach genome as a proxy for cherry, this places the endoPGs and the *F-M* locus, ~ 8 Mbp distal to the *qP-FF4.1*. In contrast to peach, no studies in cherry have associated endoPG with flesh firmness, nor were any endoPG genes been identified in the *qP-FF4.1* region. Peach and cherry also differ in their ethylene requirement for ripening. Peach is a climacteric fruit meaning that it has a strong requirement for ethylene to ripen, while cherry is a non-climacteric fruit. Taken together, these results suggest that the genetic control of fruit firmness in cherry evolved separately from that of peach. This conclusion is consistent with *qP-FF4.1* being associated with domesticated and bred germplasm.

### QTL hotspot of *qP-FF4.1* region

The scope of the work presented herein is limited to fruit firmness; however, the *qP-FF4.1* region is an important QTL “hotspot” for cherry breeders because major QTL for other traits map to this region. LG 4 loci for two phenology traits, bloom and maturity date, have been conserved across multiple *Prunus* species^27^. For bloom time, the major locus named *Lb*, was first reported in almond by Ballester *et al*.^28^ and subsequently identified in multiple *Prunus* species^29–32^. In sour cherry, the peak peach genome v2.0 position for the bloom time QTL on chromosome 4 was ~10.8 Mbp^33^. For maturity date, a major QTL termed *qMD4.1*, was identified first in peach^34,35^ and subsequently in cherry^36,37^. The most likely candidate gene for the peach QTL *qMD4.1* is ppa008301m, which is believed to be an NAC transcription factor. It maps to ~11.106 Mbp on the peach genome sequence v1.0^38^ which is equivalent to ~11.117 Mbp on the peach genome sequence v2.0^39^. In a recent study, Isuzugawa *et al*.^36^ found that two sweet cherry candidate genes, homologous to the NAC transcription factors identified in peach, also mapped within the maturity date QTL on LG 4. In addition, QTL for fruit weight and soluble solids content have also been reported in this *qP-FF4.1* region in peach^34,35^. An analysis of maturity date for the ‘Fercer’ × ‘X’ population used in our study identified a QTL that explained on average 50% of the phenotypic variance for maturity time^37^. This QTL was detected in the same region as the one for firmness; however, the peak detected with the multi-year analysis was at 36.1 cM, which is almost 2 cM downstream from the peak for the firmness QTL.

The clustering of these QTLs is in agreement with the correlations observed in the populations studied among the two fruit traits: firmness and maturity date. Indeed, within the ‘Fercer’ × ‘X’ population, early maturing individuals bear smaller and softer cherries than the late maturing ones. In the RosBREED germplasm, a prior study of this germplasm identified a QTL for maturity date that overlapped with *qP-FF4.1*^40^. In this prior study, ss490552928 was associated with maturity date with the ‘A’ and ‘B’ alleles associated with “early” and “late” maturity, respectively; however, one of the haplotypes that contained the ‘A’ allele was associated with late maturity^40^. A similar result was obtained from the INRA sweet cherry germplasm collection population where the ‘A’ for ss490552928, was also associated with early maturity and soft fruit. The phenomenon of early maturing cultivars which tend to be softer than late maturing ones could probably result from a developmental impossibility of having a firm fruit in a short period of time between blooming and maturity. This would be in particular the case of cultivar ‘Early Burlat’, which has one of the shortest developmental periods between blooming and maturity. Hence, the recent method of developing sweet cherry cultivars which come to maturity at the same period as ‘Early Burlat’, but exhibit a significantly higher firmness, was to use genitors with an extra-early blooming time^41^. In sour cherry, none of the individuals evaluated had the genotype BBBB for ss490552928; therefore, the ‘A’ allele was always present. This is consistent with “soft” fruit alleles for this locus being dominant to “firm” fruit alleles. In sour cherry, bloom and maturity time are also correlated, however all individuals whether early or late maturing have soft fruit compared to the firm fruit associated with bred sweet cherries.

The multiple QTLs in the *qP-FF4.1* region should be taken into consideration when performing breeding selection in both sweet and sour cherry. Hence, it is of utmost importance to disentangle the genetic determinism of the traits’ variation within this QTL; in particular, it would be helpful for breeders to know whether maturity date and firmness are controlled by the same pleiotropic locus or by two closely linked genes. Fine mapping initiatives might be conducted in order to search for recombinants within this narrow genetic interval. More specifically, using cultivars such as ‘Early Burlat’ and ‘Fercer’ might be highly informative. Indeed, the predicted diplotypes for both cultivars are H3H6 and H1H2, respectively; that is, each would have one “firm” and one “soft” haplotype. However, ‘Fercer’ is known to be a significantly firmer cultivar as compared to ‘Early Burlat’. Multi-year data from INRA indicate a mean firmness, as measured by Durofel, of 67 and 49 for ‘Fercer’ and ‘Early Burlat’, respectively. This shows the complexity of the genetic determinism of fruit firmness, as already demonstrated by Campoy *et al*.^6^ and might suggest as well the existence of epistatic interactions. To test the hypothesis that maturity time and firmness are controlled by distinct genes in this LG 4 “hotspot”, breeders will need to produce very large progeny populations when crossing ‘Early Burlat’ with other firmer but also later ripening cultivars in order to obtain recombinants between the two hypothesized closely linked genes. Finally, the fact that among the founders used in modern breeding, the haplotype H3 was only found in ‘Early Burlat’ agrees with the ‘originality’ of this cultivar in terms of developmental cycle; as already stated, ‘Early Burlat’ has a rather intermediate bloom time but is one of the earliest maturing cultivars.

### Candidate genes controlling fruit firmness

The available sweet cherry genome sequence provided the opportunity to identify agronomically important candidate genes for *qP-FF4.1*^42^. In our study, the identification of candidate genes was employed across species including sweet cherry, peach and tomato. Peach and sweet cherry are closely related *Prunus* species and share a high level of synteny^43^. Therefore, prior to the publication of the sweet cherry genome sequence, the peach genome was used as a proxy for candidate gene prediction in sweet cherry^6,27^. Tomato was included as fruit firmness has been extensively studied in this species^44,45^, and like cherry and peach, tomato is a fleshy carpel. Although fleshy fruits are physiologically classified as climacteric (tomato and peach) and non-climacteric (cherry), these fruits share some common characteristics such as the role of plant hormones and their interplay related to changes in firmness during fruit softening^19,44,46^. For example, all fruits appear to respond to abscisic acid (ABA) and ethylene; but, in non-climacteric fruit, even if ABA has a more dominant role, the fruit still exhibit characteristics of ethylene-dependent ripening^19^. Moreover, recent studies seem to indicate that the classification of fruits as either climacteric or non-climacteric is an oversimplification^44^. Some fruits, like melons, can display both climacteric and non-climacteric behaviors^47^ while kiwi fruit can display non-climacteric behaviors in the first stage of ripening and climacteric behaviors and in the second stage of ripening^48^. The fruit softening process involves the physiological modification of the cell wall and during the ripening phase of the fruit, plant hormones play important roles in this process^49^. Therefore, genes related to plant cell wall metabolism or various hormone signaling pathways were considered as candidates in this study. Much of tissue firmness work in tomato also focused on characterizing the potential role of cell wall–modifying genes and the transcription factors involved in hormone signaling pathways^11,23^. Although a cross-species strategy could help identify more candidate genes for fruit firmness, it should be noted that mechanisms controlling firmness in these three species are possibly different. For example, endoPG, the major enzyme associated with softening in peach, was not identified in the *qP-FF4.1* region.

Among the candidate genes identified, an expansin gene was considered as the most promising candidate for several reasons. Firstly, expansin genes have been thought to contribute to fruit softening by weakening noncovalent interactions between cellulose microfibrils and hemicellulose components^50^. In tomato, expansin genes have been shown to be associated with fruit ripening and firmness^51,52^. Secondly, the candidate expansin gene in sweet cherry has sequence homology to the expansin gene *PcEXP4* previously reported to be upregulated during tissue softening in sour cherry^16^. Thirdly, the expansin gene in sweet cherry was predicted to contain two functional domains; one of them was commonly found in pollen allergens which were proposed as cell wall-loosening agents to induce extension of the plant cell wall^53^. Lastly, this gene is very close to the peak of the *qP-FF4.1* region. Among other candidate genes, transcription factors, such as NAC domain protein, MADS-box protein and Squamosa promoter-Binding Protein could also play roles in regulating fruit firmness as they have been shown to be involved in fruit ripening process^21,23,54^. However, future work is needed to fine map this region and ultimately identify and characterize the genes and their alleles that underlie these QTL. The haplotypes and their germplasm sources described in this study provide a genetic framework for this future discovery.

In conclusion, we identified a major QTL for fruit firmness in three sweet cherry populations that is associated with domesticated and bred germplasm. As all commercial sweet cherry cultivars must meet consumer demands for firm fruit, knowledge of the desirable alleles at this QTL would be targets for marker-assisted breeding. For example, it would be especially useful to select against “soft” alleles in cases where wild germplasm is being use as breeding parents. Candidate genes for this fruit firmness QTL were proposed; however, future fine mapping and transcriptomic analysis is needed to enable the identification of the underlying gene(s).

## Methods

### Plant materials

Three sweet cherry populations were used in this study. These were: (1) an INRA bi-parental F_1_ population, (2) the INRA sweet cherry germplasm collection, and (3) RosBREED (www.rosbreed.org) pedigreed population. The INRA bi-parental F_1_ population consisted of a progeny of 67 individuals derived from a cross between cultivar ‘Fercer’ and an unknown parent called ‘X’. The INRA sweet cherry germplasm collection, maintained by INRA’s *Prunus* Genetic Resources Center at Bourran, France, included 193 sweet cherry accessions collected from France and other 15 countries of America, Asia and Europe^55^. RosBREED pedigreed populations consisted of a set of 65 elite sweet cherry and mazzard clones, and 463 unselected F_1_ seedlings from 86 crosses from the Washington State University sweet cherry breeding program (Table S2). The germplasm of RosBREED pedigreed population, spanning six generations, was considered representative of U.S. public breeding germplasm for this crop^24^. For sour cherry, a total of 338 individuals including parents, ancestors and offspring from five bi-parental F_1_ populations, were used. These individuals were grown at the Michigan State University Clarksville Research Station, Clarksville, Michigan. A detailed description of these sour cherry plant materials is presented in Cai *et al*.^33^.

### Phenotyping and phenotype modeling

Fruit were harvested from the field when ripe based on a subjective assessment of skin color, texture and taste^56,57^, placed in coolers for transport back to the laboratory, and evaluated the same day. Fruit firmness (g/mm^2^) was evaluated using different methods for INRA’s populations and RosBREED’s sweet and sour cherry pedigreed populations. For the two INRA populations (F_1_ population and the sweet cherry germplasm collection), fruit firmness was measured using a Durofel (Setop Giraud Technologie, Cavaillon, France) texture analyzer on the day of harvest. A 3-mm probe was applied at two points on the fruit equator, the movement of the probe was recorded and the average of the two measures on ten fruits was used. Data were collected from seven years (2009–2013, 2015 and 2016) for the F_1_ population and two years for the germplasm collection (2014 and 2015). For the RosBREED sweet cherry and sour cherry individuals, fruit firmness was measured from 25 fruits that were at room temperature using the compression test of BioWorks FirmTech 2 (Wamego, KS, USA). Compression was performed from the fruit cheek and with the stems still on the fruit. The mean value of 25 measures were used. The data for the sweet and sour cherry individuals were collected for two years (sweet cherry, 2011 and 2012; sour cherry, 2011 and 2013). Since different methods were used to measure firmness in the RosBREED and INRA populations, the data for these populations were analyzed separately.

The phenotypic data were analyzed using SAS version 9.13 (SAS Institute Inc.) and the model PROC MIXED was used to obtain the variance components. PROC CORR was performed to calculate the correlation coefficients of fruit firmness among different years. Broad-sense heritability (*H*^2^) was calculated using estimates for the individuals based on the following random linear model:

$${{\rm{Y}}}_{{\rm{ij}}}=\mu +{{\rm{y}}}_{{\rm{i}}}+{{\rm{g}}}_{{\rm{j}}}+{{\rm{e}}}_{{\rm{ij}}}$$

where Y_ij_ is the phenotypic value of the j^th^ individual in i^th^ year; µ is the mean value of fruit firmness; y_i_ is the random effect of the i^th^ year on the phenotype; g_j_ is the random genotypic effect of j^th^ individual; and e_ij_ is the model residual. *H*^2^ was calculated using the following equation: *H*^2^ = σ^2^_g_/(σ^2^_g_ + σ^2^_e_/n), where σ^2^_g_ is the genetic variance, σ^2^_e_ is the residual error, and n is the number of years.

### Genotyping and genetic map

All individuals from the three sweet cherry populations were genotyped with the RosBREED Illumina Infinium cherry SNP array of 5,696 SNP markers^58^ and SNP genotypes were scored using the Genotyping Module of GenomeStudio Data Analysis software^59^. For the ‘Fercer’ × ‘X’ F_1_ population, a total of 724 SNP markers were polymorphic and segregating in this population. A linkage map was constructed using JoinMap 4.0^60^ and Kosambi’s mapping function was used to convert recombination frequency into map distance. The two resulting parental maps consisted of 110 and 87 SNP markers, polymorphic for ‘Fercer’ and ‘X’, respectively. For the INRA sweet cherry germplasm collection, marker data curation was described in Campoy *et al*.^55^. A total of 1,215 SNP markers were retained after removing the following four SNP types: (1) SNPs failing to generate clear genotype clusters; (2) SNPs with missing genotypes greater than 5%; (3) SNPs showing high distortion for Hardy-Weinberg equilibrium (>0.0001); and (4) SNPs with minor allele frequencies lower than 5%. For RosBREED pedigreed population, marker data curation was described in Cai *et al*.^24^. A total of 1,617 SNPs were identified as robust markers and therefore used for QTL analysis. Genetic positions for these markers were determined by aligning and integrating these physical positions (based on Peach Genome v2.0)^39^ with the sweet cherry ‘Regina’ × ‘Lapins’ SNP linkage map^61^. The sour cherry plant materials were also genotyped using the RosBREED Illumina Infinium cherry SNP array^58^. The generation of the sour cherry genetic data, including haplotype reconstruction was described in Cai *et al*.^33^.

### QTL analysis

QTL analyses were performed for all three sweet cherry populations using different mapping softwares. For ‘Fercer’ × ‘X’, QTL mapping was carried out using MultiQTL v2.6 software with the multiple interval mapping (MIM) approach used (MultiQTL Ltd, Haifa, Israel, 2005, www.multiQTL.com). Different types of analyses were performed for single year and multiple years, respectively. Each year was first analyzed independently in order to examine the stability of the QTLs. An analysis combining all years was performed using the multiple environment option with increase of the accuracy of the QTL detection. The detailed QTL mapping methodology was as described in Castède *et al*.^27^. The graphical presentation of QTL locations on the linkage groups was obtained using the MapChart software version 2.2^62^.

A genome-wide association analysis was done for the INRA sweet cherry germplasm collection. This analysis tested the association between fruit firmness and the SNP markers on the chromosome where the firmness QTL was located. A total of 1215 SNP markers across the sweet cherry genome were used in the analysis and the SNPs associated with fruit firmness were identified according to a mixed linear model (MLM) using the software TASSEL version 5.2.61^63^. Corrections for population structure and kinship were adopted in the model. Population structure was described in the study of Campoy *et al*.^55^. The relative kinship matrix was calculated using SPAGeDi^64^. The genome-wide significance cutoff was set at 4 × 10^−5^ (0.05/1215).

QTL analysis in RosBREED sweet cherry pedigreed population was done using FlexQTL software that is designed for use with multiple pedigree-connected families^65^. Markov Chain Monte Carlo (MCMC) simulation, implemented in FlexQTL software, was applied to obtain samples from the joint posterior distribution of the model. A total of 1,000 samples (500,000 iterations with thinning value of 500) were stored for each simulation and then used for statistical inference. The inference on the number of QTLs was based on a pairwise comparison of models differing from each other by one QTL. The Bayes factor parameters (2lnBF) were interpreted as non-significant (0–2), positive (2–5), strong (5–10) or decisive (>10) evidence for the presence of QTL. The inference on the QTL position was based on posterior intensity and the inference on the QTL contribution was based on the posterior mean estimates of the QTL effect size. Both additive and dominant genetic models were tested using a maximum number of 10 QTLs. Prior number of QTL was set to 1 or 3 and genome-wide analyses were performed twice for each prior using different seed numbers to test the robustness of the analysis.

### Haplotype (diplotype) analysis

Haplotypes (i.e. alleles) for the fruit firmness QTL were identified for the INRA F_1_ population and the RosBREED sweet and sour cherry pedigreed germplasm. Five SNPs covering the consensus QTL region identified from the sets of plant materials were chosen for haplotype construction. Phased SNP marker information was obtained for each individual from the RosBREED sweet cherry germplasm from the FlexQTL output. Haplotypes were assigned using the PediHaplotyper software^66^. The SNP phasing for the sour cherry haplotypes was described in Cai *et al*.^33^. Unique haplotypes were named from H1 to H16 randomly. Statistical analyses for the association between haplotype (diplotype) and fruit firmness were performed using the software R version 3.1.3^67^. QTL genotypes (*QQ*, *Qq*, *qq*) predicted by FlexQTL were used to deduce if a haplotype was associated with soft or firm fruit. As ‘Q’ is assigned to the higher phenotypic value, in this case increased firmness, *QQ*, *Qq*, and *qq* genotypes correspond to two “firm” alleles, one “firm” and one “soft” allele, and two “soft” alleles, respectively. Haplotypes were also deduced to be associated with soft or firm fruit based on comparison of diplotypes.

### *In silico* Candidate Genes

The list of genes within the QTL interval and their functional annotations in sweet cherry were obtained from the sweet cherry genome^42^ available on the Genome Database of Rosaceae website (GDR, https://www.rosaceae.org). The corresponding predicted cherry protein sequences obtained from GDR were blasted against the National Center for Biotechnology Information (NCBI) database to obtain gene ontology terms using BLASTP in the program Blast2GO^68^ with an E-value cutoff of 0.001. The sequences of the genes within the QTL interval were also blasted to the peach genome v2.0^39^ available at GDR and the tomato genome ITAG 2.40^69^ available at Sol genomics Network (https://solgenomics.net), and their best gene matches and annotations were extracted. Overall, three annotations (sweet cherry, peach and tomato) and gene ontology results from Blast2GO were considered for the identification of candidate genes for fruit firmness. Candidate genes considered were those genes predicted to be involved in plant cell wall metabolism or various hormone (Ethylene, Brassinosteroid, Auxin, Gibberellin, and ABA) signaling pathways associated with fruit ripening. A circular plot of the mapping and candidate gene data was prepared with the Circos plotting tool^70^. Alignment and analysis of the expansin genes was performed with ClustalW v2.1 multiple sequences alignment tool. A dendrogram of the four genes was constructed using the Jukes-Cantor genetic distance model, and the neighbor-joining tree building method within the GeneiousV.11.1.4 GUI software^71,72^.

## Supplementary information

Supplementary Information

Dataset1

## Data Availability

Genotypic data for the INRA bi-parental population and RosBREED pedigreed populations is available at the Genome Database for Rosaceae (www.rosaceae.org/publication_datasets) under accession number tfGDR1037. All other data generated or analyzed during this study are included within this article and its Supplementary Information Files.
