# Supplementary material for: A fruit firmness QTL identified on linkage group 4 in sweet cherry (Prunus avium L.) is associated with domesticated and bred germplasm
Source: Sci Rep. 2019 Mar 21;9:5008. doi: 10.1038/s41598-019-41484-8 (PMC6428808; doi:10.1038/s41598-019-41484-8)
Supplement: Supplementary file 1 — Supplementary Information [file 41598_2019_41484_MOESM1_ESM.pdf]

A fruit firmness QTL identified on linkage group 4 in sweet cherry (*Prunus avium* L.) is associated with domesticated and bred germplasm

Lichun Cai[1], José Quero-García[2], Teresa Barreneche[2], Elisabeth Dirlewanger[2], Christopher Saski[3], Amy Iezzoni[1,*]

[1]Department of Horticulture, Michigan State University, East Lansing, MI, 48824, USA

[2]UMR 1332 BFP, INRA, Université de Bordeaux, F-33140 Villenave d'Ornon, France

[3]Department of Plant and Environmental Sciences, Clemson University, Clemson, SC, 29634, USA

[*] Correspondence: iezzoni@msu.edu

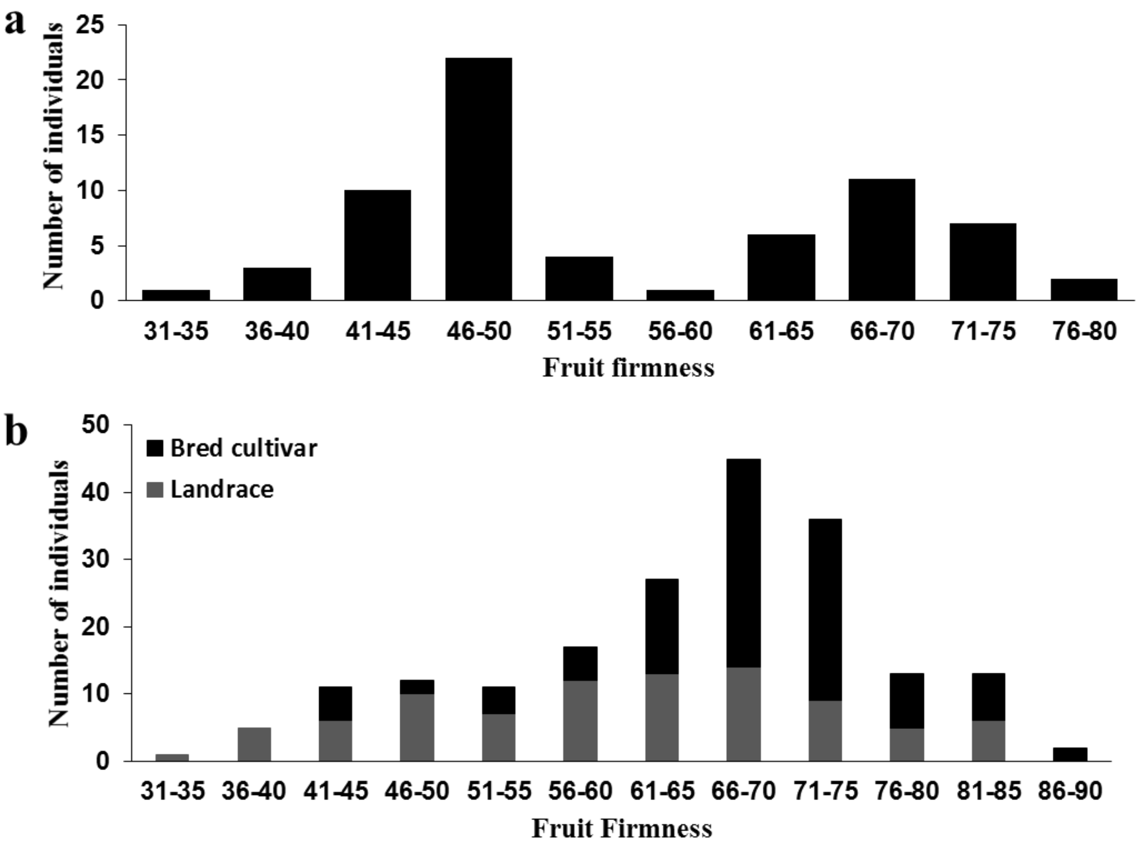

Fig. S1. Frequency distributions of fruit firmness for INRA's two sweet cherry populations: (**a**) Mean values of fruit firmness from six years (2009 to 2013, and 2015 to 2016) for the INRA 'Fercer' × 'X' F_1 population; and (**b**) Mean values of fruit firmness from two years (2014 and 2015) for the INRA sweet cherry germplasm collection.

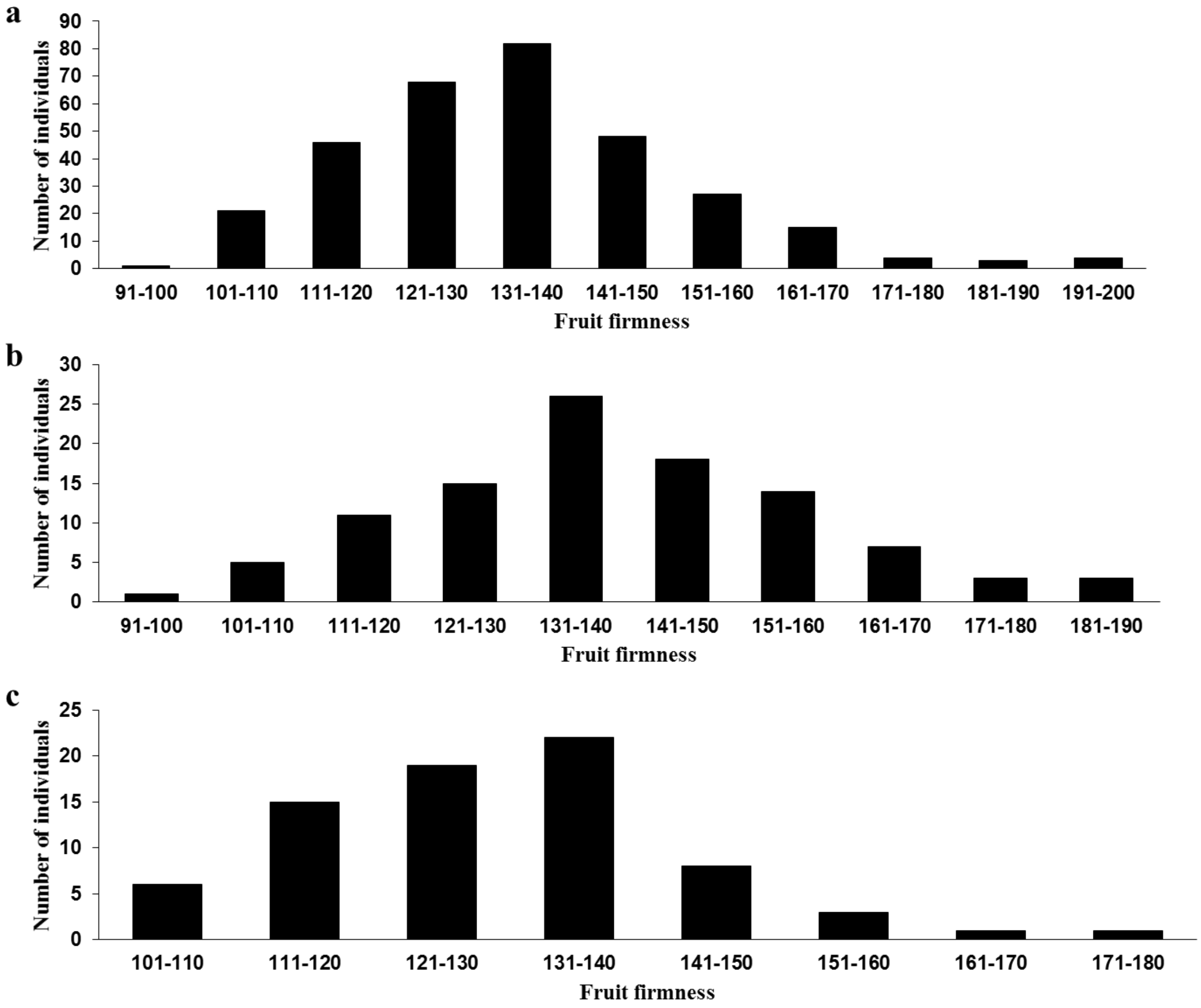

Fig. S2. Frequency distributions of fruit firmness for sour cherry populations: (**a**) all the sour cherry individuals including the five F_1 populations; (**b**) 'M172' × 25-02-29 F_1 population; (**c**) 'Balaton' × 'Surefire' F_1 population.

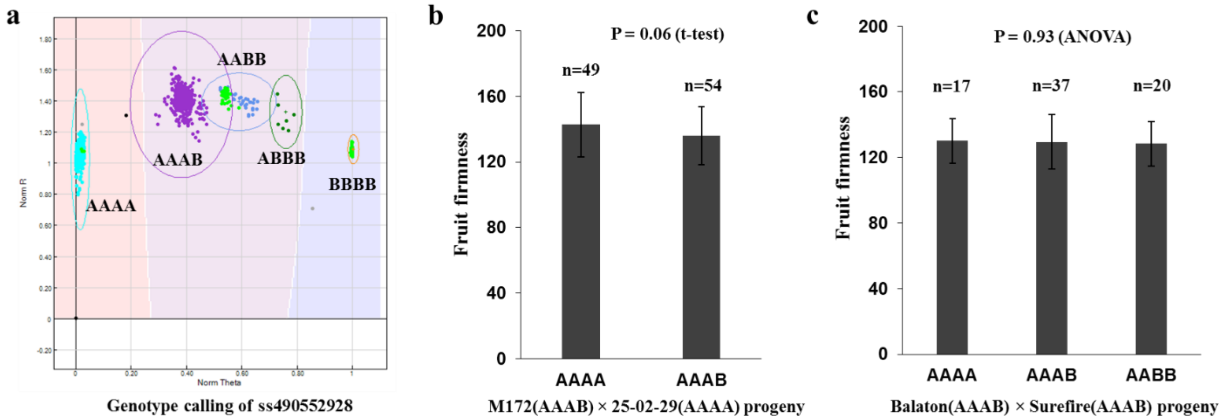

Fig. S3. Genotypes for the SNP marker ss490552928 in sour cherry and a comparison of the genotype means for fruit firmness. (**a**) Image of Genome Studio clustering for this SNP marker. Bright green identifies sweet cherry individuals used as references to identify the AA, AB and BB clusters that correspond to the AAAA, AABB and BBBB tetraploid genotypes, respectively. All other colors correspond to sour cherries that fall within the AAAA, AAAB, AABB and AAAB clusters. No sour cherry individuals are BBBB for SNP marker ss490552928. (**b**) Comparison of fruit firmness (g/mm^2) between different SNP genotypes (AAAA and AAAB) in M172 × 25-02-29 progeny. (**c**) Comparison of fruit firmness (g/mm^2) among different SNP genotypes (AAAA, AAAB and AABB) in 'Balaton' × 'Surefire' progeny.

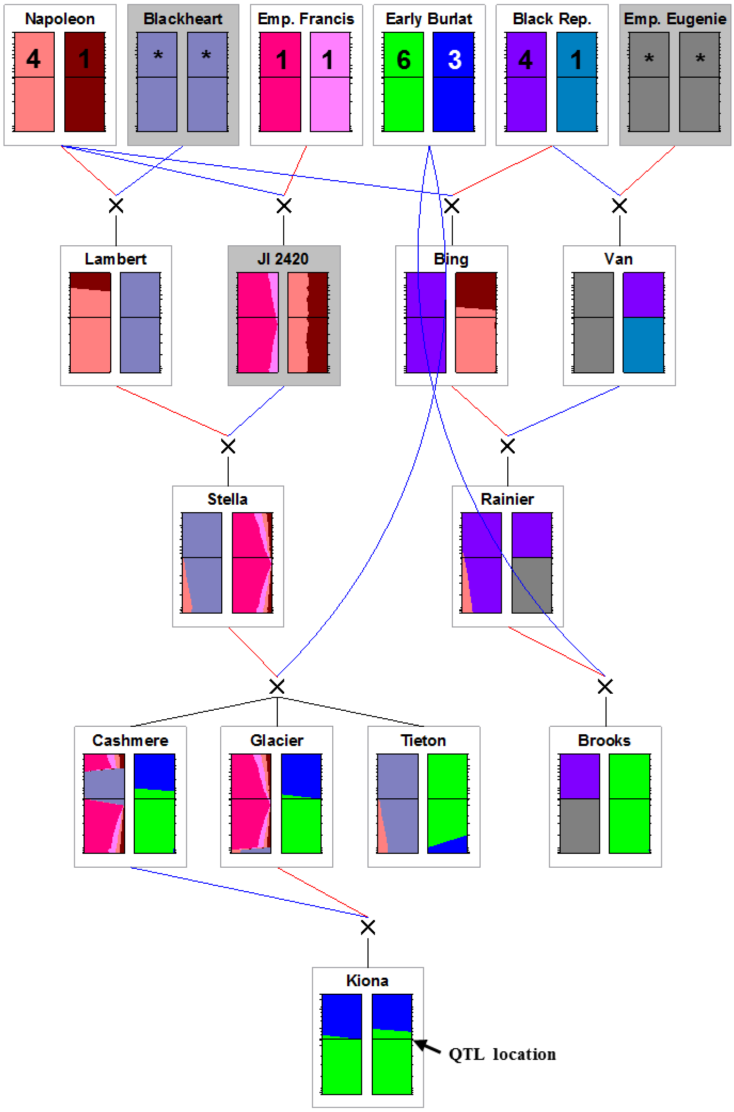

Fig. S4. Identity by descent (IBD) of ‘Early Burlat’ derived progeny (‘Cashmere’, ‘Glacier’, ‘Tieton’, ‘Brooks’, and ‘Kiona’) from the Washington State University sweet cherry breeding program. Each rectangle in an individual represents one of the two copies of chromosome 4, where each color represents a distinct founder chromosome. Within each individual, the one on the left was inherited from the mother (red line) and the one on the right from the father (blue line). White background color indicates an individual was genotyped and grey indicates not genotyped. For founders not genotyped (‘Empress Eugenie’ and ‘Blackheart’), the two founder

homologs were assigned the same color. The width of the color at each position along the chromosome indicates the IBD probability that the corresponding founder allele is present in this individual. The black line in each rectangle indicates the location of *qP-FF4.1* to illustrate that all the ‘Early Burlat’ offspring inherited the H6 haplotype. Known founder alleles (haplotypes) for *qP-FF4.1* are indicated. The images were drawn using Pedimap software[1].

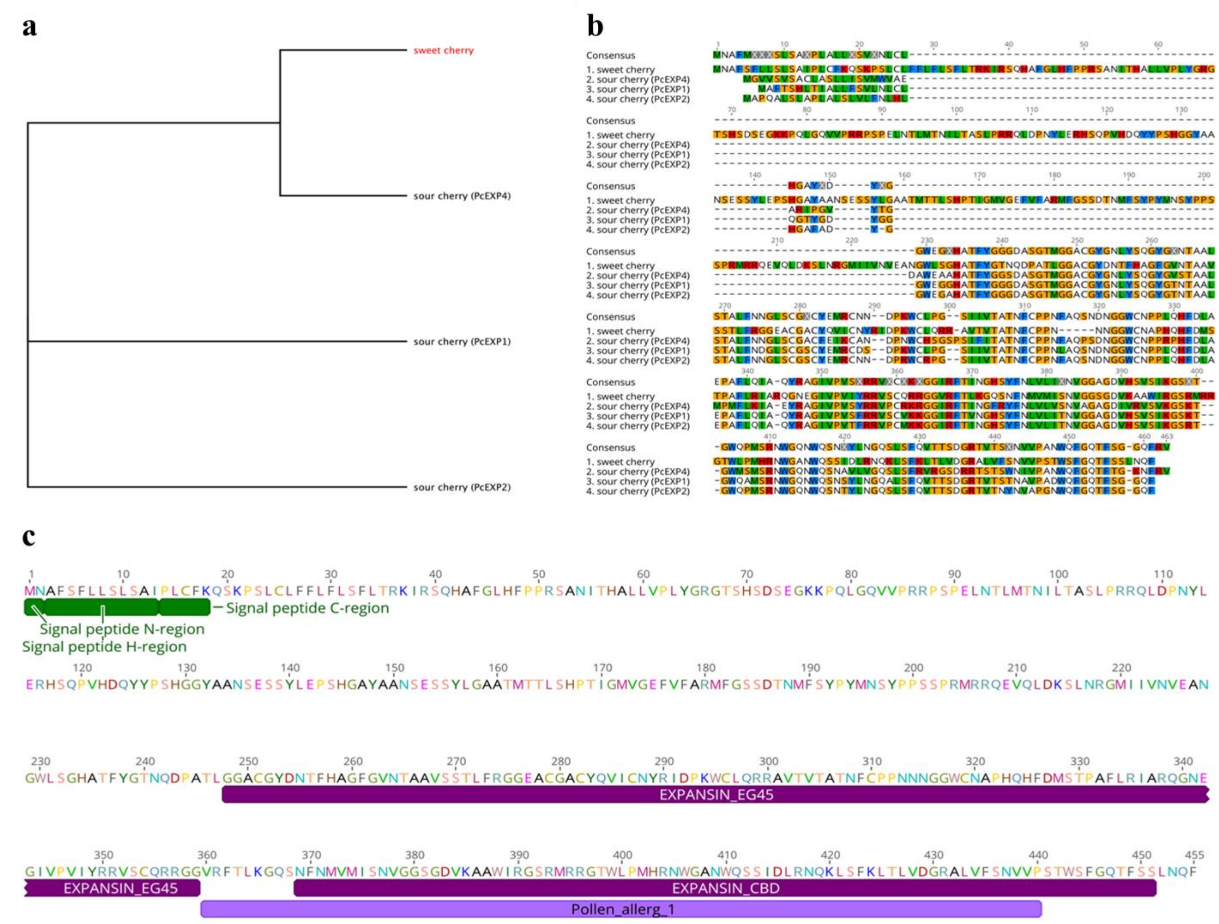

Fig. S5. (**a**) Neighbor-joining tree of the candidate sweet cherry expansin gene relative to alignment to three sour cherry expansin genes from Yoo *et al.*[2]. (**b**). An amino acid alignment of the sweet cherry expansin gene with the other three sour cherry expansin genes. **(c).** Functional domain prediction of the expansin gene.

Table S1. All the QTLs identified for fruit firmness in INRA's 'Fercer' × 'X' population and RosBREED pedigreed population.

Population	Year	LG[a]	LOD[b]	CI (cM)[c]	Peak (cM)	d[d]	PVE (%)[e]
INRA 'Fercer' × 'X'	2009	X4	10.7	[28.4;38.0]	33.2	-17.6	62.8
	2010	X4	23.3	[30.2;38.0]	34.1	-26.3	82.4
	2011	X4	18.1	[30.0;40.1]	35.0	-22.5	70.2
	2012	X4	22.4	[31.4;39.1]	35.2	-22.9	78.3
	2013	X4	11.3	[24.9;42.8]	33.9	-18.7	54.0
	2015	X4	25.9	[30.5;38.0]	34.2	-25.7	84.6
	2016	X4	17.6	[30.8;39.0]	34.9	-21.7	70.8
	MY	F4	20.6	[10.3;67.7]	39.0	-12.1	20.1
		F5	14.3	[76.0;88.4]	82.2	8.2	10.3
		F8	9.8	[50.8;80.6]	70.0	5.8	6.0
		X4	125.3	[33.1;36.0]	34.5	-21.9	70.2
		X6	11.8	[47.3;64.8]	56.1	-3.9	3.0
**Population**	**Year**	**LG**	**BF[f]**	**Interval (cM)**	**Peak (cM)**	**Effect[g]**	**PVE (%)**
RosBREED	2011	2	6.5	[29;70]	47.0	23.4	7.4
	2011	4	4.8	[31;47]	33.0	48.0	16.4
	2011	8	3.9	[9;51]	29.0	48.3	24.6
	2012	1	5.2	[32;54]	45.0	20.0	4.3
	2012	2	4.3	[36;55]	45.0	17.0	1.8
	2012	4	32.1	[31;37]	33.0	83.0	83.5
	2012	6	4.8	[68;88]	73.0	21.5	4.4

[a] Linkage group (LG) of 'X' (X); linkage group (LG) of 'Fercer' (F)

[b] Logarithm of odds ratio (LOD)

[c] Confidence interval (CI)

[d] Difference between the two homozygotes at the marker loci (d)

[e] Percentage of variation explained by the QTL (PVE)

[f] Significance was presented by Bayes Factor (BF)

[g] Additive effect

Table S2. RosBREED germplasm along with the *qP-FF4.1* FlexQTL genotype predictions and diplotypes.

Name	Maternal parent	Paternal parent	Type	*QQ*	*Qq*	*qq*	***qP-FF4.1* diplotypes**
AA	PMR-1	Rainier	Landrace or bred cultivar	1	0	0	H4H4
Ambrunes	*	*	Landrace or bred cultivar	1	0	0	H1H1
BB	PMR-1	Rainier	Landrace or bred cultivar	1	0	0	H4H4
Benton	Stella	Moreau	Landrace or bred cultivar	1	0	0	H1H4
Bertiolle	*	*	Landrace or bred cultivar	0.97	0.02	0	H5H5
Bing	Black Republican	Napoleon	Landrace or bred cultivar	0.97	0.03	0	H4H4
Black Republican	Napoleon	F_BR	Landrace or bred cultivar	0.76	0.24	0	H1H4
Blackheart	*	*	Landrace or bred cultivar	0.76	0.24	0	No genotypic data
Brooks	Rainier	Early Burlat	Landrace or bred cultivar	1	0	0	H4H6
Cashmere	Stella	Early Burlat	Landrace or bred cultivar	1	0	0	H4H6
CC	PMR-1	Rainier	Landrace or bred cultivar	1	0	0	H4H4
Chelan	Stella	Moreau	Landrace or bred cultivar	1	0	0	H1H1
Chinook	Bing	Gil-Peck	Landrace or bred cultivar	0.74	0.25	0.01	H4H4
Cowiche	PC 7147-4	PC 7146-11	Landrace or bred cultivar	1	0	0	H4H4
Cristobalina	*	*	Landrace or bred cultivar	0	1	0	H1H9
DD	PMR-1	Rainier	Landrace or bred cultivar	1	0	0	H4H4
Dzherlo	*	*	Landrace or bred cultivar	0.76	0.24	0	H1H4
Early Burlat	*	*	Landrace or bred cultivar	0.8	0.2	0	H3H6
EE	PMR-1	Rainier	Landrace or bred cultivar	1	0	0	H4H4
Emperor Francis	*	*	Landrace or bred cultivar	1	0	0	H1H1
Empress Eugenie	*	*	Landrace or bred cultivar	0.74	0.26	0	No genotypic data
GG	PMR-1	Rainier	Landrace or bred cultivar	1	0	0	H4H4
Giant	*	*	Landrace or bred cultivar	0.58	0.35	0.07	No genotypic data
Gil-Peck	Napoleon	Giant	Landrace or bred cultivar	0.74	0.25	0.01	H4H4
Glacier	Stella	Early Burlat	Landrace or bred cultivar	1	0	0	H1H6
Governor Wood	*	*	Landrace or bred cultivar	0.55	0.38	0.06	No genotypic data
Hedelfingen	*	*	Landrace or bred cultivar	0.75	0.25	0	No genotypic data
Index	Stella	Bing	Landrace or bred cultivar	1	0	0	H1H4

JI 2420	Emperor Francis	Napoleon	Landrace or bred cultivar	0.86	0.14	0	No genotypic data
JJ	PMR-1	Rainier	Landrace or bred cultivar	1	0	0	H4H4
Kiona	Glacier	Cashmere	Landrace or bred cultivar	1	0	0	H6H6
Kordia	Schneiders	*	Landrace or bred cultivar	1	0	0	H2H5
Krupnoplodnaya	*	*	Landrace or bred cultivar	1	0	0	H4H5
Lambert	Napoleon	Blackheart	Landrace or bred cultivar	0.97	0.03	0	H4H4
Lapins	Stella	Van	Landrace or bred cultivar	1	0	0	H1H4
MIM 17	*	*	Wild cherry	0	0	1	H8H10
MIM 23	*	*	Wild cherry	0	0	1	H11H12
Moreau	*	*	Landrace or bred cultivar	0	1	0	H1H2
Napoleon	*	*	Landrace or bred cultivar	0.73	0.26	0.01	H1H4
Newstar	Van	Stella	Landrace or bred cultivar	1	0	0	H1H4
NY 54	*	*	Wild cherry	0	0.05	0.95	H13H13
Olympus	Lambert	Van	Landrace or bred cultivar	1	0	0	H4H4
P8-79	*	*	Landrace or bred cultivar	0.98	0.02	0	No genotypic data
PC 7146-11	Stella	Moreau	Landrace or bred cultivar	0.17	0.83	0	No genotypic data
PC 7146-16	Stella	Moreau	Landrace or bred cultivar	1	0	0	H1H1
PC 7147-4	Stella	*	Landrace or bred cultivar	1	0	0	No genotypic data
PMR-1	*	*	Landrace or bred cultivar	1	0	0	H4H4
Rainier	Bing	Van	Landrace or bred cultivar	1	0	0	H4H4
Regina	Schneiders	Rube	Landrace or bred cultivar	1	0	0	H4H5
Rube	*	*	Landrace or bred cultivar	0.73	0.27	0	No genotypic data
Satonishiki	Governor Wood	Napoleon	Landrace or bred cultivar	0.57	0.35	0.08	H1H10
Schmidt	*	*	Landrace or bred cultivar	0.58	0.36	0.06	H1H1
Schneiders	*	*	Landrace or bred cultivar	0.75	0.25	0	H5H7
Selah	P8-79	Stella	Landrace or bred cultivar	1	0	0	H1H4
Stella	Lambert	JI 2420	Landrace or bred cultivar	1	0	0	H1H4
Summit	Van	*	Landrace or bred cultivar	0.99	0.01	0	H4H4
Sunburst	Summit	*	Landrace or bred cultivar	0.97	0.02	0.01	H4H4
Sweetheart	Van	Lapins	Landrace or bred cultivar	1	0	0	H1H1
Tieton	Stella	Early Burlat	Landrace or bred cultivar	1	0	0	H4H6

Ulster	Napoleon	Gil-Peck	Landrace or bred cultivar	0.74	0.25	0.01	H4H4
Van	Empress Eugenie	Black Republican	Landrace or bred cultivar	1	0	0	H1H4
Venus	Hedelfingen	Windsor	Landrace or bred cultivar	1	0	0	H1H4
Vic	Bing	Schmidt	Landrace or bred cultivar	0.74	0.25	0.01	H1H4
Vittoria	*	*	Landrace or bred cultivar	0.89	0.11	0	H1H1
Windsor	*	*	Landrace or bred cultivar	0.77	0.23	0	H1H4
23-01-11	NY 54	Emperor Francis	Wild × Cultivar F_1 offspring	0	1	0	H1H13
23-01-47	NY 54	Emperor Francis	Wild × Cultivar F_1 offspring	0	1	0	H1H13
23-02-34	NY 54	Emperor Francis	Wild × Cultivar F_1 offspring	0	1	0	H1H13
23-02-46	NY 54	Emperor Francis	Wild × Cultivar F_1 offspring	0.04	0.96	0	H1H13
23-03-13	NY 54	Emperor Francis	Wild × Cultivar F_1 offspring	0	1	0	H1H13
23-03-23	NY 54	Emperor Francis	Wild × Cultivar F_1 offspring	0	1	0	H1H13
23-03-24	NY 54	Emperor Francis	Wild × Cultivar F_1 offspring	0	1	0	H1H13
23-03-28	NY 54	Emperor Francis	Wild × Cultivar F_1 offspring	0	1	0	H1H13
23-03-40	NY 54	Emperor Francis	Wild × Cultivar F_1 offspring	0	1	0	H1H13
23-03-48	NY 54	Emperor Francis	Wild × Cultivar F_1 offspring	0	1	0	H1H13
23-03-56	NY 54	Emperor Francis	Wild × Cultivar F_1 offspring	0	1	0	H1H13
23-05-17	NY 54	Emperor Francis	Wild × Cultivar F_1 offspring	0	1	0	H1H13
23-06-04	NY 54	Emperor Francis	Wild × Cultivar F_1 offspring	0	1	0	H1H13
23-06-23	NY 54	Emperor Francis	Wild × Cultivar F_1 offspring	0	1	0	H1H13
23-07-26	NY 54	Emperor Francis	Wild × Cultivar F_1 offspring	0	1	0	H1H13
23-08-24	NY 54	Emperor Francis	Wild × Cultivar F_1 offspring	0	1	0	H1H13
23-08-45	NY 54	Emperor Francis	Wild × Cultivar F_1 offspring	0	1	0	H1H13
23-08-55	NY 54	Emperor Francis	Wild × Cultivar F_1 offspring	0	1	0	H1H13
9814-025	Bing	PMR-1	Landrace or bred cultivar	1	0	0	H4H4
9814-026	Bing	PMR-1	Landrace or bred cultivar	1	0	0	H4H4
9814-028	Bing	PMR-1	Landrace or bred cultivar	1	0	0	H4H4
9814-055	Bing	PMR-1	Landrace or bred cultivar	1	0	0	H4H4
9814-062	Bing	PMR-1	Landrace or bred cultivar	1	0	0	H4H4
9814-063	Bing	PMR-1	Landrace or bred cultivar	0.97	0.03	0	H4H4
9814-068	Bing	PMR-1	Landrace or bred cultivar	0.97	0.03	0	H4H4

9814-071	Bing	PMR-1	Landrace or bred cultivar	0.97	0.03	0	H4H4
9814-076	Bing	PMR-1	Landrace or bred cultivar	1	0	0	H4H4
9814-078	Bing	PMR-1	Landrace or bred cultivar	0.97	0.03	0	H4H4
9814-103	Bing	PMR-1	Landrace or bred cultivar	0.97	0.03	0	H4H4
9814-105	Bing	PMR-1	Landrace or bred cultivar	1	0	0	H4H4
9816-005	Rainier	PMR-1	Landrace or bred cultivar	1	0	0	H4H4
9816-023	Rainier	PMR-1	Landrace or bred cultivar	1	0	0	H4H4
9816-041	Rainier	PMR-1	Landrace or bred cultivar	1	0	0	H4H4
9816-042	Rainier	*	Landrace or bred cultivar	0.79	0.21	0	H4H7
9816-059	Rainier	PMR-1	Landrace or bred cultivar	1	0	0	H4H4
9816-078	Van	PMR-1	Landrace or bred cultivar	1	0	0	H4H4
9816-083	Rainier	PMR-1	Landrace or bred cultivar	1	0	0	H4H4
9816-103	Rainier	PMR-1	Landrace or bred cultivar	1	0	0	H4H4
9816-105	Rainier	PMR-1	Landrace or bred cultivar	1	0	0	H4H4
9816-106	Rainier	PMR-1	Landrace or bred cultivar	1	0	0	H4H4
9816-112	Rainier	PMR-1	Landrace or bred cultivar	1	0	0	H4H4
9819-005	PMR-1	Van	Landrace or bred cultivar	1	0	0	H1H4
9819-031	PMR-1	Van	Landrace or bred cultivar	1	0	0	H1H4
9819-034	PMR-1	Bing	Landrace or bred cultivar	1	0	0	H4H4
9819-038	PMR-1	Van	Landrace or bred cultivar	1	0	0	H4H4
FR10T098	PMR-1	Moreau	Landrace or bred cultivar	0	1	0	H2H4
FR10T099	PMR-1	Tieton	Landrace or bred cultivar	1	0	0	H4H4
FR10T101	PMR-1	Moreau	Landrace or bred cultivar	1	0	0	H1H4
FR11T015	Rainier	Regina	Landrace or bred cultivar	1	0	0	H4H4
FR11T016	Rainier	Regina	Landrace or bred cultivar	1	0	0	H4H5
FR11T017	Rainier	Regina	Landrace or bred cultivar	1	0	0	H4H4
FR11T018	Rainier	Regina	Landrace or bred cultivar	1	0	0	H4H5
FR11T019	Rainier	Regina	Landrace or bred cultivar	1	0	0	H4H5
FR11T025	Rainier	Regina	Landrace or bred cultivar	1	0	0	H4H5
FR11T026	Rainier	Regina	Landrace or bred cultivar	1	0	0	H4H5
FR11T027	Rainier	Regina	Landrace or bred cultivar	1	0	0	H4H5

FR11T028	Rainier	Regina	Landrace or bred cultivar	1	0	0	H4H5
FR11T029	Rainier	Regina	Landrace or bred cultivar	1	0	0	H4H4
FR11T030	Rainier	Regina	Landrace or bred cultivar	1	0	0	H4H4
FR11T031	Rainier	Regina	Landrace or bred cultivar	1	0	0	H4H4
FR11T032	Rainier	Regina	Landrace or bred cultivar	1	0	0	H4H5
FR12T001	Lapins	Moreau	Landrace or bred cultivar	0.49	0.51	0	Recombination within QTL region
FR12T003	Lapins	Moreau	Landrace or bred cultivar	1	0	0	H1H1
FR12T004	Lapins	Moreau	Landrace or bred cultivar	1	0	0	H1H4
FR12T006	Lapins	Moreau	Landrace or bred cultivar	0	1	0	H1H2
FR12T009	Lapins	Moreau	Landrace or bred cultivar	1	0	0	H1H4
FR12T011	Lapins	Moreau	Landrace or bred cultivar	1	0	0	H1H1
FR12T012	Lapins	Moreau	Landrace or bred cultivar	0	1	0	H1H2
FR12T055	Lapins	Venus	Landrace or bred cultivar	1	0	0	H1H4
FR12T056	Lapins	Venus	Landrace or bred cultivar	1	0	0	H1H4
FR12T057	Lapins	Venus	Landrace or bred cultivar	1	0	0	H1H1
FR12T058	Lapins	Venus	Landrace or bred cultivar	1	0	0	H1H1
FR12T060	Lapins	Venus	Landrace or bred cultivar	1	0	0	H4H4
FR12T063	Lapins	Venus	Landrace or bred cultivar	1	0	0	H1H4
FR12T066	Lapins	Venus	Landrace or bred cultivar	1	0	0	H4H4
FR12T067	Lapins	Venus	Landrace or bred cultivar	1	0	0	H1H4
FR12T068	Lapins	Venus	Landrace or bred cultivar	1	0	0	H1H4
FR12T069	Lapins	Venus	Landrace or bred cultivar	1	0	0	H1H4
FR12T071	Lapins	Venus	Landrace or bred cultivar	1	0	0	H1H4
FR12T072	Lapins	Venus	Landrace or bred cultivar	1	0	0	H1H4
FR12T073	Lapins	Venus	Landrace or bred cultivar	1	0	0	H1H4
FR12T074	Lapins	Venus	Landrace or bred cultivar	1	0	0	H1H4
FR12T076	Lapins	Venus	Landrace or bred cultivar	1	0	0	H1H1
FR12T077	Selah	Moreau	Landrace or bred cultivar	1	0	0	H1H4
FR12T078	Lapins	Venus	Landrace or bred cultivar	1	0	0	H1H1
FR12T080	Selah	Venus	Landrace or bred cultivar	1	0	0	H1H4
FR12T081	Selah	Venus	Landrace or bred cultivar	1	0	0	H1H1

FR12T091	Rainier	Moreau	Landrace or bred cultivar	0	1	0	H2H4
FR12T093	Rainier	Moreau	Landrace or bred cultivar	0	1	0	H2H4
FR12T094	Rainier	Moreau	Landrace or bred cultivar	1	0	0	H1H4
FR13T004	Sweetheart	BB	Landrace or bred cultivar	1	0	0	H1H4
FR13T058	Selah	Moreau	Landrace or bred cultivar	0	1	0	H2H4
FR13T060	Selah	Moreau	Landrace or bred cultivar	0	1	0	H1H2
FR13T061	Selah	Moreau	Landrace or bred cultivar	1	0	0	H1H4
FR13T071	Selah	*	Landrace or bred cultivar	0.98	0.02	0	H4H4
FR14T012	Benton	Selah	Landrace or bred cultivar	1	0	0	H1H1
FR14T056	Cowiche	Regina	Landrace or bred cultivar	1	0	0	H4H4
FR14T057	Cowiche	Regina	Landrace or bred cultivar	1	0	0	H4H5
FR14T060	Cowiche	Regina	Landrace or bred cultivar	1	0	0	H4H5
FR14T075	Cowiche	Regina	Landrace or bred cultivar	1	0	0	H4H4
FR14T076	Cowiche	Regina	Landrace or bred cultivar	1	0	0	H4H5
FR14T078	Cowiche	Regina	Landrace or bred cultivar	1	0	0	H4H5
FR14T079	Cowiche	Regina	Landrace or bred cultivar	1	0	0	H4H4
FR14T080	Cowiche	Regina	Landrace or bred cultivar	1	0	0	H4H4
FR14T081	Cowiche	Regina	Landrace or bred cultivar	1	0	0	H4H5
FR14T082	Cowiche	Regina	Landrace or bred cultivar	1	0	0	H4H5
FR14T084	Cowiche	Regina	Landrace or bred cultivar	1	0	0	H4H4
FR14T086	Cowiche	Regina	Landrace or bred cultivar	1	0	0	H4H4
FR14T100	Cowiche	Regina	Landrace or bred cultivar	1	0	0	H4H5
FR16T001	Benton	Ambrunes	Landrace or bred cultivar	1	0	0	H1H1
FR16T012	Kiona	*	Landrace or bred cultivar	0.76	0.24	0	H4H6
FR16T024	Kordia	Van	Landrace or bred cultivar	1	0	0	H2H4
FR16T040	Benton	Benton	Landrace or bred cultivar	1	0	0	H1H4
FR16T041	Bing	Regina	Landrace or bred cultivar	0.97	0.03	0	H4H4
FR17T006	Bing	Regina	Landrace or bred cultivar	1	0	0	H4H5
FR17T051	Cowiche	Summit	Landrace or bred cultivar	1	0	0	H4H4
FR17T052	Cowiche	Summit	Landrace or bred cultivar	0.99	0.01	0	H4H4
FR17T053	Cowiche	Summit	Landrace or bred cultivar	1	0	0	H4H4

FR17T054	Cowiche	Summit	Landrace or bred cultivar	1	0	0	H4H4
FR17T055	Cowiche	Summit	Landrace or bred cultivar	0.99	0.01	0	H4H4
FR17T059	Cowiche	Summit	Landrace or bred cultivar	1	0	0	H4H4
FR17T060	Cowiche	Summit	Landrace or bred cultivar	1	0	0	H4H4
FR17T061	Cowiche	Summit	Landrace or bred cultivar	0.99	0.01	0	H4H4
FR17T062	Cowiche	Summit	Landrace or bred cultivar	1	0	0	H4H4
FR17T063	Lapins	Schneiders	Landrace or bred cultivar	0.75	0.25	0	H4H7
FR17T066	Bing	Regina	Landrace or bred cultivar	0.97	0.03	0	H4H5
FR17T073	Benton	Ambrunes	Landrace or bred cultivar	1	0	0	H1H4
FR17T080	Benton	Ambrunes	Landrace or bred cultivar	1	0	0	H1H4
FR17T082	Benton	Regina	Landrace or bred cultivar	1	0	0	H4H5
FR18T017	Kordia	Bing	Landrace or bred cultivar	0.98	0.02	0	H4H5
FR18T018	Kordia	Regina	Landrace or bred cultivar	1	0	0	H5H5
FR18T019	Kordia	Bing	Landrace or bred cultivar	1	0	0	H2H4
FR18T020	Kordia	Regina	Landrace or bred cultivar	1	0	0	Recombination within QTL region
FR18T041	Kordia	Regina	Landrace or bred cultivar	1	0	0	H2H5
FR18T042	Kordia	Regina	Landrace or bred cultivar	1	0	0	H4H5
FR18T043	Sweetheart	Krupnoplodnaya	Landrace or bred cultivar	1	0	0	H1H5
FR18T044	Kordia	Regina	Landrace or bred cultivar	1	0	0	H2H4
FR18T045	Kordia	Bing	Landrace or bred cultivar	1	0	0	H4H5
FR18T047	Kordia	Bing	Landrace or bred cultivar	1	0	0	H2H4
FR18T048	Kordia	Bing	Landrace or bred cultivar	1	0	0	H2H4
FR18T052	Kordia	Van	Landrace or bred cultivar	1	0	0	H2H4
FR18T071	Rainier	Benton	Landrace or bred cultivar	1	0	0	H1H4
FR18T073	Rainier	Benton	Landrace or bred cultivar	1	0	0	H4H4
FR18T074	Rainier	Benton	Landrace or bred cultivar	1	0	0	H4H4
FR18T076	Rainier	Benton	Landrace or bred cultivar	1	0	0	H1H4
FR19T066	Selah	*	Landrace or bred cultivar	0.73	0.27	0	H4H4
FR19T069	Selah	Krupnoplodnaya	Landrace or bred cultivar	1	0	0	H4H5
FR19T070	Selah	Krupnoplodnaya	Landrace or bred cultivar	1	0	0	H1H5
FR19T071	Selah	Krupnoplodnaya	Landrace or bred cultivar	1	0	0	H1H5

FR19T072	Selah	Krupnoplodnaya	Landrace or bred cultivar	1	0	0	H4H4
FR19T073	Selah	Selah	Landrace or bred cultivar	1	0	0	H1H1
FR19T075	Selah	Selah	Landrace or bred cultivar	1	0	0	H1H1
FR19T076	Selah	Krupnoplodnaya	Landrace or bred cultivar	1	0	0	H1H4
FR19T078	Selah	Krupnoplodnaya	Landrace or bred cultivar	1	0	0	H4H5
FR1T027	Lapins	Regina	Landrace or bred cultivar	1	0	0	H4H5
FR1T028	Lapins	Regina	Landrace or bred cultivar	1	0	0	H1H4
FR1T029	Lapins	Regina	Landrace or bred cultivar	1	0	0	H1H5
FR1T030	Lapins	Regina	Landrace or bred cultivar	1	0	0	H1H4
FR1T031	Lapins	Regina	Landrace or bred cultivar	1	0	0	H1H5
FR1T032	Lapins	Regina	Landrace or bred cultivar	1	0	0	H1H4
FR1T033	Lapins	Bing	Landrace or bred cultivar	1	0	0	H1H4
FR1T035	Lapins	Regina	Landrace or bred cultivar	1	0	0	H4H4
FR1T036	Lapins	Regina	Landrace or bred cultivar	1	0	0	H1H4
FR1T037	Lapins	Regina	Landrace or bred cultivar	1	0	0	H4H4
FR1T038	Lapins	Regina	Landrace or bred cultivar	1	0	0	H1H5
FR1T039	Lapins	Regina	Landrace or bred cultivar	1	0	0	H1H4
FR1T040	Lapins	Regina	Landrace or bred cultivar	1	0	0	H1H5
FR1T041	Lapins	Regina	Landrace or bred cultivar	1	0	0	H4H4
FR1T042	Lapins	Regina	Landrace or bred cultivar	1	0	0	H4H5
FR1T050	Lapins	Kordia	Landrace or bred cultivar	1	0	0	H1H2
FR1T054	Lapins	Regina	Landrace or bred cultivar	1	0	0	H1H5
FR1T056	Lapins	Regina	Landrace or bred cultivar	1	0	0	H1H5
FR1T065	Lapins	Regina	Landrace or bred cultivar	1	0	0	H1H4
FR1T066	Lapins	Regina	Landrace or bred cultivar	1	0	0	H4H4
FR1T068	Lapins	Regina	Landrace or bred cultivar	1	0	0	H1H5
FR1T069	Lapins	Regina	Landrace or bred cultivar	1	0	0	H1H4
FR1T070	Lapins	Regina	Landrace or bred cultivar	1	0	0	H1H4
FR1T073	Lapins	Regina	Landrace or bred cultivar	1	0	0	H1H5
FR1T074	Lapins	Regina	Landrace or bred cultivar	1	0	0	H4H5
FR20T045	Cowiche	Regina	Landrace or bred cultivar	1	0	0	H4H5

FR20T047	Cowiche	Chelan	Landrace or bred cultivar	1	0	0	H1H4
FR21T003	Selah	Selah	Landrace or bred cultivar	1	0	0	H1H4
FR21T037	Bing	Regina	Landrace or bred cultivar	0.97	0.03	0	H4H5
FR21T040	Bing	Regina	Landrace or bred cultivar	0.99	0.01	0	H4H5
FR21T063	Selah	Krupnoplodnaya	Landrace or bred cultivar	1	0	0	H1H4
FR22T001	Cowiche	Bertiolle	Landrace or bred cultivar	0.99	0.01	0	H4H5
FR22T002	Cowiche	Bertiolle	Landrace or bred cultivar	0.99	0.01	0	H4H5
FR22T003	Cowiche	Rainier	Landrace or bred cultivar	1	0	0	H4H4
FR22T004	Cowiche	Tieton	Landrace or bred cultivar	1	0	0	H4H4
FR22T005	Cowiche	*	Landrace or bred cultivar	0.93	0.07	0	H4H4
FR22T006	Cowiche	Chelan	Landrace or bred cultivar	1	0	0	H1H4
FR22T007	Cowiche	Cristobalina	Landrace or bred cultivar	1	0	0	H1H4
FR23T010	Benton	Benton	Landrace or bred cultivar	1	0	0	H4H4
FR23T011	Benton	Benton	Landrace or bred cultivar	1	0	0	H1H4
FR23T065	BB	MIM 23	Wild × Cultivar F_1 offspring	0	1	0	H4H12
FR23T067	BB	MIM 23	Wild × Cultivar F_1 offspring	0	1	0	H4H12
FR23T068	BB	MIM 23	Wild × Cultivar F_1 offspring	0	1	0	H4H8
FR23T069	BB	MIM 23	Wild × Cultivar F_1 offspring	0	1	0	H4H12
FR23T070	BB	MIM 23	Wild × Cultivar F_1 offspring	0	1	0	H4H12
FR23T072	BB	MIM 23	Wild × Cultivar F_1 offspring	0	1	0	H4H11
FR23T073	BB	MIM 23	Wild × Cultivar F_1 offspring	0	1	0	Recombination within QTL region
FR23T089	BB	MIM 17	Wild × Cultivar F_1 offspring	0	1	0	H4H8
FR23T090	BB	MIM 17	Wild × Cultivar F_1 offspring	0	1	0	H4H8
FR23T091	BB	MIM 17	Wild × Cultivar F_1 offspring	0	1	0	H4H10
FR23T093	BB	MIM 17	Wild × Cultivar F_1 offspring	0	1	0	H4H10
FR23T095	BB	MIM 17	Wild × Cultivar F_1 offspring	0	1	0	H4H10
FR23T097	BB	MIM 17	Wild × Cultivar F_1 offspring	0	1	0	H4H8
FR23T112	Kiona	Cristobalina	Landrace or bred cultivar	0	1	0	H6H9
FR23T113	Kiona	Cristobalina	Landrace or bred cultivar	0	1	0	H6H9
FR23T114	Kiona	Cristobalina	Landrace or bred cultivar	0	1	0	H6H9
FR23T115	Kiona	Cristobalina	Landrace or bred cultivar	0.87	0.13	0	H1H6

FR23T116	Kiona	Cristobalina	Landrace or bred cultivar	1	0	0	H1H6
FR23T119	Kiona	Cristobalina	Landrace or bred cultivar	0	1	0	H6H9
FR23T120	Kiona	Cristobalina	Landrace or bred cultivar	0	1	0	H6H9
FR23T123	Kiona	Cristobalina	Landrace or bred cultivar	0	1	0	H6H9
FR23T124	Kiona	Cristobalina	Landrace or bred cultivar	0.12	0.88	0	H6H9
FR23T127	Cowiche	Vittoria	Landrace or bred cultivar	1	0	0	H1H4
FR23T128	Cowiche	Index	Landrace or bred cultivar	1	0	0	H1H4
FR23T129	Cowiche	Vittoria	Landrace or bred cultivar	1	0	0	H1H4
FR23T130	Cowiche	Vittoria	Landrace or bred cultivar	1	0	0	H1H4
FR23T131	Cowiche	Vittoria	Landrace or bred cultivar	1	0	0	H1H4
FR23T132	Cowiche	*	Landrace or bred cultivar	0.75	0.25	0	H4H4
FR23T133	Cowiche	*	Landrace or bred cultivar	0.8	0.2	0	H4H4
FR24T064	Benton	*	Landrace or bred cultivar	0.45	0.55	0	H1H1
FR24T066	Benton	Benton	Landrace or bred cultivar	1	0	0	H1H4
FR24T067	Benton	Benton	Landrace or bred cultivar	1	0	0	H1H4
FR24T068	Benton	Dzherlo	Landrace or bred cultivar	0.99	0.01	0	H1H4
FR24T069	Benton	Dzherlo	Landrace or bred cultivar	0.76	0.24	0	H1H4
FR24T070	Benton	*	Landrace or bred cultivar	0.77	0.23	0	H1H7
FR24T071	Benton	Dzherlo	Landrace or bred cultivar	1	0	0	H1H4
FR24T072	Benton	Dzherlo	Landrace or bred cultivar	0.76	0.24	0	H4H4
FR24T073	Benton	*	Landrace or bred cultivar	0.73	0.27	0	H4H4
FR24T077	Benton	Dzherlo	Landrace or bred cultivar	1	0	0	H1H4
FR25T081	Benton	Vittoria	Landrace or bred cultivar	1	0	0	H1H1
FR25T084	Benton	Vittoria	Landrace or bred cultivar	1	0	0	H1H4
FR25T085	Benton	Benton	Landrace or bred cultivar	1	0	0	H4H4
FR25T086	Benton	Vittoria	Landrace or bred cultivar	0.98	0.02	0	H1H1
FR25T090	Benton	Vittoria	Landrace or bred cultivar	1	0	0	H1H1
FR25T092	Benton	Benton	Landrace or bred cultivar	1	0	0	H4H4
FR25T093	Benton	Vittoria	Landrace or bred cultivar	1	0	0	H1H1
FR25T097	Benton	*	Landrace or bred cultivar	0.75	0.25	0	H4H4
FR25T098	Benton	*	Landrace or bred cultivar	0.76	0.24	0	H1H4

FR26T028	Bing	*	Landrace or bred cultivar	0.74	0.25	0.01	H1H4
FR26T030	Bing	Bertiolle	Landrace or bred cultivar	0.94	0.06	0	H4H5
FR26T031	Bing	Bertiolle	Landrace or bred cultivar	0.96	0.04	0	H4H5
FR26T033	Bing	Bertiolle	Landrace or bred cultivar	0.99	0.01	0	H4H5
FR26T034	Bing	Bertiolle	Landrace or bred cultivar	0.98	0.02	0	H4H5
FR26T036	Bing	Bertiolle	Landrace or bred cultivar	0.98	0.02	0	H4H5
FR26T037	Bing	Bertiolle	Landrace or bred cultivar	0.96	0.04	0	H4H5
FR26T038	Bing	Bertiolle	Landrace or bred cultivar	0.96	0.04	0	H4H5
FR26T040	Bing	Selah	Landrace or bred cultivar	1	0	0	H4H4
FR26T117	Bing	Lapins	Landrace or bred cultivar	0.97	0.03	0	H1H4
FR27T001	DD	MIM 23	Wild × Cultivar F_1 offspring	0	1	0	H4H11
FR27T002	DD	MIM 23	Wild × Cultivar F_1 offspring	0	1	0	H4H11
FR27T003	DD	MIM 23	Wild × Cultivar F_1 offspring	0	1	0	H4H11
FR27T006	DD	MIM 17	Wild × Cultivar F_1 offspring	0	1	0	H4H10
FR27T007	DD	MIM 17	Wild × Cultivar F_1 offspring	0	1	0	H4H8
FR27T008	DD	MIM 17	Wild × Cultivar F_1 offspring	0	1	0	H4H10
FR27T009	DD	MIM 17	Wild × Cultivar F_1 offspring	0	1	0	H4H9
FR27T010	DD	MIM 17	Wild × Cultivar F_1 offspring	0	1	0	H4H8
FR27T012	DD	MIM 17	Wild × Cultivar F_1 offspring	0	1	0	Recombination within QTL region
FR27T013	DD	MIM 17	Wild × Cultivar F_1 offspring	0	1	0	H4H8
FR27T014	DD	MIM 17	Wild × Cultivar F_1 offspring	0	1	0	H4H10
FR27T015	DD	MIM 17	Wild × Cultivar F_1 offspring	0	1	0	H4H8
FR27T016	DD	MIM 17	Wild × Cultivar F_1 offspring	0	1	0	H4H10
FR27T017	DD	MIM 17	Wild × Cultivar F_1 offspring	0	1	0	H4H8
FR27T018	DD	MIM 17	Wild × Cultivar F_1 offspring	0	1	0	H4H10
FR27T050	CC	Venus	Landrace or bred cultivar	1	0	0	H1H4
FR27T051	CC	Bing	Landrace or bred cultivar	0.97	0.03	0	H4H4
FR27T052	CC	Venus	Landrace or bred cultivar	1	0	0	H1H4
FR27T053	CC	MIM 23	Wild × Cultivar F_1 offspring	0	1	0	H4H12
FR27T054	CC	MIM 23	Wild × Cultivar F_1 offspring	0	1	0	H4H11
FR27T094	CC	MIM 17	Wild × Cultivar F_1 offspring	0	1	0	H4H8

FR27T096	CC	MIM 17	Wild × Cultivar F_1 offspring	0	1	0	H4H10
FR27T097	CC	MIM 17	Wild × Cultivar F_1 offspring	0	1	0	H4H8
FR27T098	CC	MIM 17	Wild × Cultivar F_1 offspring	0	1	0	Recombination within QTL region
FR27T099	CC	MIM 17	Wild × Cultivar F_1 offspring	0	1	0	H4H10
FR27T100	CC	MIM 17	Wild × Cultivar F_1 offspring	0	1	0	H4H8
FR27T101	CC	MIM 17	Wild × Cultivar F_1 offspring	0	1	0	H4H8
FR27T102	CC	MIM 17	Wild × Cultivar F_1 offspring	0	1	0	H4H8
FR28T001	DD	MIM 23	Wild × Cultivar F_1 offspring	0	1	0	H4H12
FR28T002	DD	MIM 23	Wild × Cultivar F_1 offspring	0	1	0	H4H11
FR28T099	EE	MIM 17	Wild × Cultivar F_1 offspring	0	1	0	H4H8
FR28T100	EE	MIM 17	Wild × Cultivar F_1 offspring	0	1	0	H4H8
FR28T101	EE	MIM 17	Wild × Cultivar F_1 offspring	0	1	0	H4H8
FR28T102	EE	MIM 17	Wild × Cultivar F_1 offspring	0	1	0	H4H8
FR28T104	EE	MIM 17	Wild × Cultivar F_1 offspring	0	1	0	H4H10
FR28T105	EE	MIM 17	Wild × Cultivar F_1 offspring	0	1	0	H4H10
FR28T106	EE	MIM 17	Wild × Cultivar F_1 offspring	0	1	0	H4H8
FR28T107	EE	MIM 17	Wild × Cultivar F_1 offspring	0	1	0	H4H10
FR28T108	EE	MIM 17	Wild × Cultivar F_1 offspring	0	1	0	H4H10
FR29T073	GG	MIM 23	Wild × Cultivar F_1 offspring	0	1	0	H4H11
FR29T074	GG	MIM 17	Wild × Cultivar F_1 offspring	0	1	0	H4H10
FR29T075	GG	MIM 17	Wild × Cultivar F_1 offspring	0	1	0	H4H10
FR29T076	GG	MIM 17	Wild × Cultivar F_1 offspring	0	1	0	H4H10
FR29T077	GG	MIM 17	Wild × Cultivar F_1 offspring	0	1	0	H4H8
FR29T115	EE	MIM 23	Wild × Cultivar F_1 offspring	0	1	0	H4H12
FR29T117	EE	MIM 23	Wild × Cultivar F_1 offspring	0	1	0	H4H12
FR29T118	EE	MIM 23	Wild × Cultivar F_1 offspring	0	1	0	H4H11
FR29T119	EE	MIM 23	Wild × Cultivar F_1 offspring	0	1	0	H4H11
FR29T120	EE	MIM 23	Wild × Cultivar F_1 offspring	0	1	0	H4H11
FR29T122	EE	MIM 23	Wild × Cultivar F_1 offspring	0	1	0	H4H12
FR29T124	EE	MIM 23	Wild × Cultivar F_1 offspring	0	1	0	H4H12
FR29T125	EE	MIM 23	Wild × Cultivar F_1 offspring	0	1	0	H4H12

FR29T126	EE	MIM 23	Wild × Cultivar F_1 offspring	0	1	0	H4H11
FR2T024	Selah	Sunburst	Landrace or bred cultivar	0.98	0.02	0	H4H4
FR2T030	Rainier	Sweetheart	Landrace or bred cultivar	1	0	0	H1H4
FR2T035	Lapins	Ambrunes	Landrace or bred cultivar	1	0	0	H1H4
FR2T037	Lapins	Ambrunes	Landrace or bred cultivar	1	0	0	H1H4
FR2T038	Lapins	Ambrunes	Landrace or bred cultivar	1	0	0	H1H1
FR2T040	Lapins	Ambrunes	Landrace or bred cultivar	1	0	0	H1H1
FR2T045	Lapins	Ambrunes	Landrace or bred cultivar	1	0	0	H1H4
FR2T046	Lapins	Ambrunes	Landrace or bred cultivar	1	0	0	H1H7
FR2T048	Lapins	Ambrunes	Landrace or bred cultivar	1	0	0	H1H4
FR2T049	Lapins	Ambrunes	Landrace or bred cultivar	1	0	0	H1H4
FR2T050	Lapins	Ambrunes	Landrace or bred cultivar	1	0	0	H1H1
FR2T054	Lapins	Ambrunes	Landrace or bred cultivar	1	0	0	H1H4
FR2T057	Lapins	Ambrunes	Landrace or bred cultivar	1	0	0	H1H1
FR2T058	Lapins	Ambrunes	Landrace or bred cultivar	1	0	0	H1H1
FR2T062	Lapins	Ambrunes	Landrace or bred cultivar	1	0	0	H1H1
FR2T063	Lapins	Ambrunes	Landrace or bred cultivar	1	0	0	H1H1
FR2T079	Lapins	Ambrunes	Landrace or bred cultivar	1	0	0	H1H4
FR2T080	Lapins	Ambrunes	Landrace or bred cultivar	1	0	0	H1H4
FR30T099	Lapins	Vittoria	Landrace or bred cultivar	1	0	0	H1H1
FR30T100	Lapins	Vittoria	Landrace or bred cultivar	0.9	0.1	0	H1H1
FR30T101	Lapins	Vittoria	Landrace or bred cultivar	0.97	0.03	0	H1H4
FR30T102	Lapins	Vittoria	Landrace or bred cultivar	1	0	0	H1H1
FR30T103	Lapins	Vittoria	Landrace or bred cultivar	0.97	0.03	0	H1H4
FR30T105	Lapins	Vittoria	Landrace or bred cultivar	1	0	0	H1H7
FR30T106	Lapins	Vittoria	Landrace or bred cultivar	1	0	0	H1H4
FR30T107	Lapins	Vittoria	Landrace or bred cultivar	1	0	0	H1H4
FR30T108	Lapins	Vittoria	Landrace or bred cultivar	1	0	0	H1H1
FR30T109	Lapins	Vittoria	Landrace or bred cultivar	1	0	0	H1H1
FR31T007	Rainier	Ambrunes	Landrace or bred cultivar	1	0	0	H1H4
FR31T008	Rainier	Ambrunes	Landrace or bred cultivar	1	0	0	H1H4

FR31T009	Rainier	Ambrunes	Landrace or bred cultivar	1	0	0	H1H4
FR31T010	Rainier	Dzherlo	Landrace or bred cultivar	1	0	0	H1H4
FR31T011	Rainier	Ambrunes	Landrace or bred cultivar	1	0	0	H1H4
FR31T012	Rainier	Ambrunes	Landrace or bred cultivar	1	0	0	H1H4
FR31T013	Rainier	Ambrunes	Landrace or bred cultivar	1	0	0	H1H4
FR31T014	Rainier	Dzherlo	Landrace or bred cultivar	1	0	0	H1H4
FR31T016	Rainier	Ambrunes	Landrace or bred cultivar	1	0	0	H1H4
FR31T121	Lapins	Vittoria	Landrace or bred cultivar	1	0	0	H1H1
FR32T087	Rainier	Bertiolle	Landrace or bred cultivar	0.99	0.01	0	H4H5
FR32T088	Rainier	Bertiolle	Landrace or bred cultivar	0.99	0.01	0	H4H5
FR32T093	Rainier	Bertiolle	Landrace or bred cultivar	0.98	0.02	0	H4H5
FR33T001	Rainier	Cristobalina	Landrace or bred cultivar	1	0	0	H1H4
FR33T003	Rainier	Cristobalina	Landrace or bred cultivar	0.98	0.02	0	H1H4
FR33T004	Rainier	Cristobalina	Landrace or bred cultivar	1	0	0	H1H4
FR33T005	Rainier	Cristobalina	Landrace or bred cultivar	0.95	0.05	0	H1H4
FR33T006	Rainier	Cristobalina	Landrace or bred cultivar	0.92	0.08	0	H1H4
FR33T007	Rainier	Cristobalina	Landrace or bred cultivar	0	1	0	H4H9
FR33T008	Rainier	Cristobalina	Landrace or bred cultivar	0	1	0	H4H9
FR33T011	Rainier	Cristobalina	Landrace or bred cultivar	0.63	0.37	0	H4H5
FR35T101	Rainier	Dzherlo	Landrace or bred cultivar	1	0	0	H1H4
FR35T102	Rainier	Dzherlo	Landrace or bred cultivar	1	0	0	H1H4
FR35T103	Rainier	Chelan	Landrace or bred cultivar	1	0	0	H1H4
FR35T104	Rainier	Dzherlo	Landrace or bred cultivar	0.91	0.09	0	H4H5
FR35T105	Rainier	Dzherlo	Landrace or bred cultivar	0.76	0.24	0	H4H4
FR35T106	Rainier	Dzherlo	Landrace or bred cultivar	1	0	0	H1H4
FR35T107	Rainier	Dzherlo	Landrace or bred cultivar	1	0	0	H1H4
FR35T108	Rainier	Ambrunes	Landrace or bred cultivar	1	0	0	H1H4
FR35T110	Rainier	Dzherlo	Landrace or bred cultivar	1	0	0	H1H4
FR38T001	Rainier	Sunburst	Landrace or bred cultivar	0.99	0.01	0	H4H4
FR38T002	Rainier	Sunburst	Landrace or bred cultivar	0.99	0.01	0	H4H4
FR38T003	Rainier	Sunburst	Landrace or bred cultivar	0.98	0.02	0	H4H4

FR38T005	Rainier	Sunburst	Landrace or bred cultivar	0.99	0.01	0	H4H4
FR38T006	Rainier	Sunburst	Landrace or bred cultivar	0.98	0.02	0	H4H4
FR3T002	Lapins	Ambrunes	Landrace or bred cultivar	1	0	0	H1H1
FR3T003	Lapins	Ambrunes	Landrace or bred cultivar	1	0	0	H1H4
FR3T004	Lapins	Ambrunes	Landrace or bred cultivar	1	0	0	H1H1
FR3T005	Lapins	Lapins	Landrace or bred cultivar	1	0	0	H1H1
FR3T007	Lapins	Ambrunes	Landrace or bred cultivar	1	0	0	H1H1
FR3T008	Lapins	Ambrunes	Landrace or bred cultivar	1	0	0	H1H4
FR3T009	Kordia	Van	Landrace or bred cultivar	1	0	0	H1H5
FR3T011	Kordia	Regina	Landrace or bred cultivar	1	0	0	H2H4
FR3T012	Kordia	Bing	Landrace or bred cultivar	0.97	0.03	0	H2H4
FR3T013	Kordia	Regina	Landrace or bred cultivar	1	0	0	H2H5
FR3T014	Kordia	Regina	Landrace or bred cultivar	1	0	0	H5H5
FR3T015	Kordia	Regina	Landrace or bred cultivar	1	0	0	H4H5
FR3T016	Kordia	Lapins	Landrace or bred cultivar	1	0	0	H4H5
FR3T017	Kordia	Bing	Landrace or bred cultivar	0.97	0.03	0	H2H4
FR3T018	Lapins	Tieton	Landrace or bred cultivar	1	0	0	H1H6
FR3T019	Lapins	Tieton	Landrace or bred cultivar	1	0	0	H1H6
FR3T020	Lapins	Tieton	Landrace or bred cultivar	1	0	0	H1H6
FR3T021	Lapins	Tieton	Landrace or bred cultivar	1	0	0	H1H6
FR3T022	Lapins	Regina	Landrace or bred cultivar	1	0	0	H4H5
FR3T023	Lapins	Tieton	Landrace or bred cultivar	1	0	0	H4H6
FR3T025	Lapins	Tieton	Landrace or bred cultivar	1	0	0	H1H4
FR3T026	Lapins	Tieton	Landrace or bred cultivar	1	0	0	H4H6
FR3T027	Lapins	Tieton	Landrace or bred cultivar	1	0	0	H5H6
FR3T028	Lapins	Tieton	Landrace or bred cultivar	1	0	0	H4H4
FR3T029	Lapins	Chelan	Landrace or bred cultivar	1	0	0	H1H1
FR3T030	Lapins	Chelan	Landrace or bred cultivar	1	0	0	H1H4
FR3T031	Lapins	Chelan	Landrace or bred cultivar	1	0	0	H1H1
FR3T032	Lapins	Chelan	Landrace or bred cultivar	1	0	0	H1H1
FR3T033	Lapins	Chelan	Landrace or bred cultivar	1	0	0	H1H4

FR3T034	Lapins	Chelan	Landrace or bred cultivar	1	0	0	H1H1
FR3T043	Lapins	Lapins	Landrace or bred cultivar	1	0	0	H1H1
FR3T044	Lapins	Chelan	Landrace or bred cultivar	1	0	0	H1H4
FR3T046	Lapins	Chelan	Landrace or bred cultivar	1	0	0	H1H1
FR3T047	Lapins	Chelan	Landrace or bred cultivar	1	0	0	H1H4
FR3T050	Lapins	Chelan	Landrace or bred cultivar	1	0	0	H1H1
FR3T056	Lapins	Chelan	Landrace or bred cultivar	1	0	0	H1H1
FR3T057	Lapins	Chelan	Landrace or bred cultivar	1	0	0	H1H4
FR3T058	Lapins	Bing	Landrace or bred cultivar	0.97	0.03	0	H4H4
FR3T070	Selah	Ambrunes	Landrace or bred cultivar	1	0	0	H1H4
FR42T001	Selah	Bertiolle	Landrace or bred cultivar	0.98	0.02	0	H4H5
FR42T003	Selah	Bertiolle	Landrace or bred cultivar	0.98	0.02	0	H4H5
FR42T005	Selah	Bertiolle	Landrace or bred cultivar	0.99	0.01	0	H1H5
FR42T006	Selah	*	Landrace or bred cultivar	0.74	0.26	0	H1H4
FR42T007	Selah	Benton	Landrace or bred cultivar	1	0	0	H1H4
FR42T008	Selah	*	Landrace or bred cultivar	0.74	0.26	0	H4H5
FR42T009	Selah	*	Landrace or bred cultivar	0.95	0.05	0	H4H4
FR42T011	Selah	Selah	Landrace or bred cultivar	1	0	0	H1H4
FR42T012	Selah	*	Landrace or bred cultivar	0.75	0.25	0	H1H4
FR42T042	Selah	*	Landrace or bred cultivar	1	0	0	H1H4
FR42T045	Selah	Selah	Landrace or bred cultivar	1	0	0	H4H4
FR42T047	Bing	Selah	Landrace or bred cultivar	0.97	0.03	0	H4H4
FR42T048	Selah	Selah	Landrace or bred cultivar	1	0	0	H1H1
FR42T049	Benton	Selah	Landrace or bred cultivar	1	0	0	H1H4
FR42T050	Benton	Selah	Landrace or bred cultivar	1	0	0	H1H4
FR47T058	Selah	Vittoria	Landrace or bred cultivar	1	0	0	H1H4
FR47T059	Selah	Vittoria	Landrace or bred cultivar	1	0	0	H1H1
FR49T083	Sweetheart	Regina	Landrace or bred cultivar	1	0	0	H1H5
FR4T029	Selah	*	Landrace or bred cultivar	1	0	0	H4H4
FR5T019	Kordia	Van	Landrace or bred cultivar	1	0	0	H1H5
FR5T021	Kordia	Van	Landrace or bred cultivar	1	0	0	H1H5

FR5T025	Kordia	Lapins	Landrace or bred cultivar	1	0	0	H2H4
FR6T097	Sweetheart	CC	Landrace or bred cultivar	1	0	0	H1H4
FR7T026	Sweetheart	CC	Landrace or bred cultivar	1	0	0	H1H4
FR8T002	PMR-1	Moreau	Landrace or bred cultivar	0.21	0.79	0	H2H4
FR8T003	PMR-1	Moreau	Landrace or bred cultivar	1	0	0	H1H4
FR8T004	PMR-1	Moreau	Landrace or bred cultivar	0	1	0	H2H4
FR8T005	PMR-1	Moreau	Landrace or bred cultivar	0	1	0	H2H4
FR8T006	PMR-1	Moreau	Landrace or bred cultivar	1	0	0	H1H4
FR8T007	PMR-1	Moreau	Landrace or bred cultivar	1	0	0	H1H4
FR8T009	Cowiche	*	Landrace or bred cultivar	0.73	0.27	0	H4H4
FR8T010	PMR-1	Venus	Landrace or bred cultivar	1	0	0	H4H4
FR8T011	PMR-1	Venus	Landrace or bred cultivar	1	0	0	H1H4
FR8T012	PMR-1	Venus	Landrace or bred cultivar	1	0	0	H1H4
FR8T047	Sweetheart	Krupnoplodnaya	Landrace or bred cultivar	1	0	0	H1H5
FR8T050	Lapins	*	Landrace or bred cultivar	0.77	0.23	0	H1H4
FR8T053	Selah	Sunburst	Landrace or bred cultivar	0.99	0.01	0	H1H4
FR8T056	Selah	Selah	Landrace or bred cultivar	1	0	0	H1H1
FR8T057	Selah	Sunburst	Landrace or bred cultivar	0.99	0.01	0	H4H4
FR8T074	GG	*	Landrace or bred cultivar	0.98	0.02	0	H4H4
FR9T031	Lapins	Krupnoplodnaya	Landrace or bred cultivar	1	0	0	H4H5
FR9T032	Lapins	Krupnoplodnaya	Landrace or bred cultivar	1	0	0	H4H7
FR9T033	Sweetheart	Moreau	Landrace or bred cultivar	0	1	0	H1H2
FR9T034	Sweetheart	Moreau	Landrace or bred cultivar	0	1	0	H1H2
FR9T037	DD	Lapins	Landrace or bred cultivar	1	0	0	H4H4
FR9T081	Kiona	Chelan	Landrace or bred cultivar	1	0	0	H1H6
FR9T083	Kiona	Chelan	Landrace or bred cultivar	1	0	0	H1H6
FR9T084	Kiona	Chelan	Landrace or bred cultivar	1	0	0	H1H6
FR9T085	Kiona	Chelan	Landrace or bred cultivar	1	0	0	H1H6
FR9T086	Kiona	Chelan	Landrace or bred cultivar	1	0	0	H1H6
FR9T087	Kiona	Chelan	Landrace or bred cultivar	1	0	0	H1H6
FR9T089	Kiona	Chelan	Landrace or bred cultivar	1	0	0	H1H6

FR9T095	Kiona	*	Landrace or bred cultivar	1	0	0	H4H6
FR9T096	Kiona	Chelan	Landrace or bred cultivar	1	0	0	H1H6
FR9T097	Kiona	Chelan	Landrace or bred cultivar	1	0	0	H1H6
FR9T098	Kiona	Chelan	Landrace or bred cultivar	1	0	0	H1H6

Table S3. Frequencies and founder contributions for *qP-FF4.1* haplotypes in RosBREED sweet cherry germplasm and for eight sour cherry cultivars. 'Fercer' and 'X' are highlighted in red.

Haplotype	Frequency in sweet cherry germplasm (%)	Founder germplasm contributor(s)
H1	28.2	EmperorFrancis, Napoleon, Moreau, Ambrunés, BlackRepublican, Windsor, Schmidt, Cristobalina, Dzherlo, SatoNishiki, Vittoria, Fercer, X
H2	1.8	Moreau, Kordia, Fercer
H3	0.9	EarlyBurlat, X
H4	49.1	Napoleon, Lambert, BlackRepublican, Windsor, Cowiche, Dzherlo, Krupnoplodnaya, PMR-1,
H5	5.4	Bertiolle, Schneiders, Kordia, Krupnoplodnaya
H6	6.4	Early Burlat
H7	0.9	Schneiders
H8	0.9	MIM 17
H9	0.9	Cristobalina
H10	1.8	MIM 17
H11	0.9	MIM 23
H12	0.9	MIM 23
H13	1.8	NY 54
H14	0.0	*
H15	0.0	*
H16	0.0	*
**Haplotype**	**Frequency in eight sour cherry individuals**	**Individual (s) exhibiting the haplotype**
H1	3.1	M172
H2	0.0	*
H3	46.9	25-02-29, M172, Montmorency, 25-14-20, Balaton, Surefire, Rheinische Schattenmorelle, Englaise Timpurii
H4	0.0	*
H5	0.0	*
H6	0.0	*
H7	0.0	*

H8	3.1	25-02-29
H9	0.0	*
H10	12.5	25-02-29, M172, Montmorency, Rheinische Schattenmorelle
H11	18.8	25-02-29, M172, 25-14-20, Balaton
H12	3.1	Surefire
H13	0.0	*
H14	3.1	Montmorency
H15	6.2	Balaton, Englaise Timpurii
H16	3.1	Englaise Timpurii

Table S4. The nine haplotypes identified for the eight sour cherry parents for the fruit firmness QTL *qP-FF4.1*. The letters assigned to the haplotypes are the designations given in Cai *et al*.[3]. The haplotype nomenclature assigned in Fig. 5 is also included in the last column.

SNP		ss490552883	ss490559054	ss490552906	ss490552912	ss490552928	
Chromosome		4	4	4	4	4	
Physical position (bp)		10,241,247	10,414,884	10,880,163	11,044,975	11,472,398	
Genetic postion (cM)		33.5	33.6	33.7	33.7	33.8	
25-02-29	***a***	B	B	B	A	A	H3
	b	B	A	B	B	A	H11
	c	A	A	*B*	B	A	H8
	d	A	B	*A*	B	A	H10
M172	***d***	A	B	A	B	A	H10
	e	B	B	B	A	A	H3
	f	B	B	A	B	B	H1
	h	B	A	B	B	A	H11
Montmorency	***a***	B	B	B	A	A	H3
	d	A	B	A	B	A	H10
	i	B	B	B	A	A	H3
	l	A	A	A	B	A	H14
25-14-20	***a***	B	B	B	A	A	H3
	g	B	B	B	A	A	H3
	h	B	A	B	B	A	H11
	j	B	A	B	B	A	H11
Balaton	***a***	B	B	B	A	A	H3
	h	B	A	B	B	A	H11
	k	B	B	B	A	A	H3

	n	A	A	B	A	B	H15
Surefire	***g***	B	B	B	A	A	H3
	i	B	B	B	A	A	H3
	k	B	B	B	A	A	H3
	o	A	A	B	B	B	H12
Rheinische Schattenmorelle	***b***	B	A	*B*	B	A	H11
	d	A	B	*A*	B	A	H10
	g	B	B	B	A	A	H3
	i	B	B	B	A	A	H3
Englaise Timpurii	***a***	B	B	B	A	A	H3
	m	B	B	B	A	A	H3
	n	A	A	B	A	B	H15
	p	A	B	B	B	B	H16

Table S5. This table is considered as a large supplementary dataset and therefore included as a separate Excel document (Supplementary Dataset).

Table S6. SNP information in the *qP-FF4.1* region. The physical positions of SNPs were based on peach genome v2.0[4].

SNP	Chromosome	Physical position (bp)	Genetic position (cM)
ss490552880	4	10156468	31.9
ss490559401	4	10195053	33.5
ss490559398	4	10195120	33.5
ss490559396	4	10195144	33.5
ss490559107	4	10236421	33.5
ss490552883	4	10241247	33.5
ss490559054	4	10414884	33.6
ss490552906	4	10880163	33.7
ss490552912	4	11044975	33.7
ss490552928	4	11472398	33.8
ss490552931	4	11520743	33.8
ss490548726	4	11661240	33.9
ss490552936	4	11661765	33.9
ss490552942	4	11956655	33.9

References

1. Voorrips, R. E., Bink, M. C. A. M. & van de Weg, W. E. Pedimap: software for the visualization of genetic and phenotypic data in pedigrees. J. Hered. **103(6)**, 903-7 (2012).
2. Yoo, S. D., Gao, Z., Cantini, C., Loescher, W. H. & van Nocker, S. Fruit ripening in sour cherry: Changes in expression of genes encoding expansins and other cell-wall-modifying enzymes. J. Amer. Soc. Hort. Sci. **128**, 16-22 (2003).
3. Cai, L. *et al.* Identification of bloom date QTLs and haplotype analysis in tetraploid sour cherry (*Prunus cerasus*). Tree Genet. Genomes **14**, 22 (2018).
4. Verde, I. *et al.* The Peach v2.0 release: high-resolution linkage mapping and deep resequencing improve chromosome-scale assembly and contiguity. BMC Genomics **18(1)**, 225 (2017).
